# Generation of PAX7 Reporter Cells to Investigate Skeletal Myogenesis from Human Pluripotent Stem Cells

**DOI:** 10.1016/j.xpro.2020.100158

**Published:** 2020-11-05

**Authors:** Haibin Xi, Courtney S. Young, April D. Pyle

**Affiliations:** 1 Department of Microbiology, Immunology, and Molecular Genetics, University of California Los Angeles, Los Angeles, CA 90095, USA; 2 Eli and Edythe Broad Center of Regenerative Medicine and Stem Cell Research, University of California Los Angeles, Los Angeles, CA 90095, USA; 3 MyoGene Bio, Los Angeles, CA 90024, USA; 4 Molecular Biology Institute, University of California Los Angeles, Los Angeles, CA 90095, USA; 5 Jonsson Comprehensive Cancer Center, University of California Los Angeles, Los Angeles, CA 90095, USA

## Abstract

This protocol describes the use of CRISPR/Cas9-mediated homology-directed recombination to construct a PAX7-GFP reporter in human pluripotent stem cells (hPSCs). PAX7 is a key transcription factor and regulator of skeletal muscle stem/progenitor cells. We obtained heterozygous knockin reporter cells and validated their PAX7 expression using both artificial activation by the CRISPR/dCas9-VPR system and physiological activation during hPSC myogenic differentiation. These cells can serve as tools for better understanding of *in vitro* hPSC myogenesis and enriching myogenic cells for downstream analysis.

For complete details on the use and execution of this protocol, please refer to Xi et al. (2017) and Xi et al. (2020).

## Highlights

• Establish reporter hPSCs for myogenic transcription factor PAX7 via CRISPR/Cas9
• Fast reporter validation by dCas9-VPR artificial activation of PAX7 promoter
• Physiological reporter validation through hPSC directed myogenic differentiation

## Graphical Abstract

**Figure:**
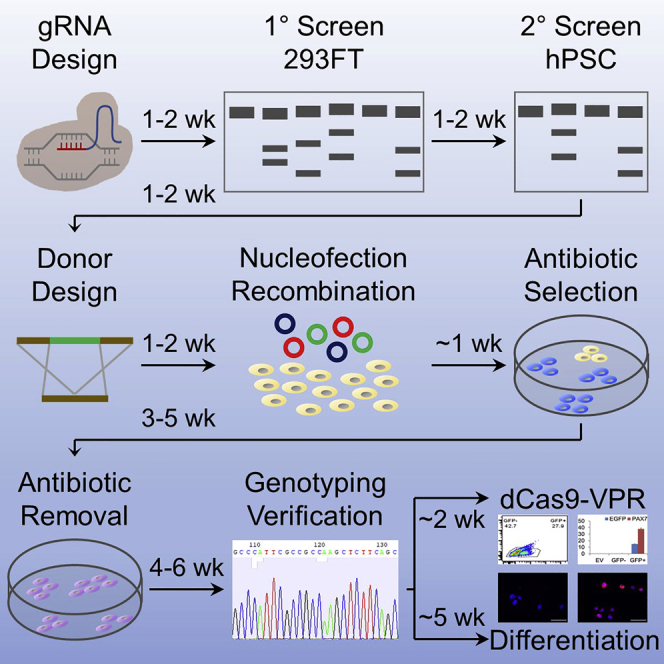

## Before You Begin

### Design of Candidate Guide RNAs (gRNAs) for Reporter Insertion

**Timing: 1 day**

1. Determine the desired transcript variant of the human *PAX7* gene to insert the reporter cassette.

**Figure 1:**
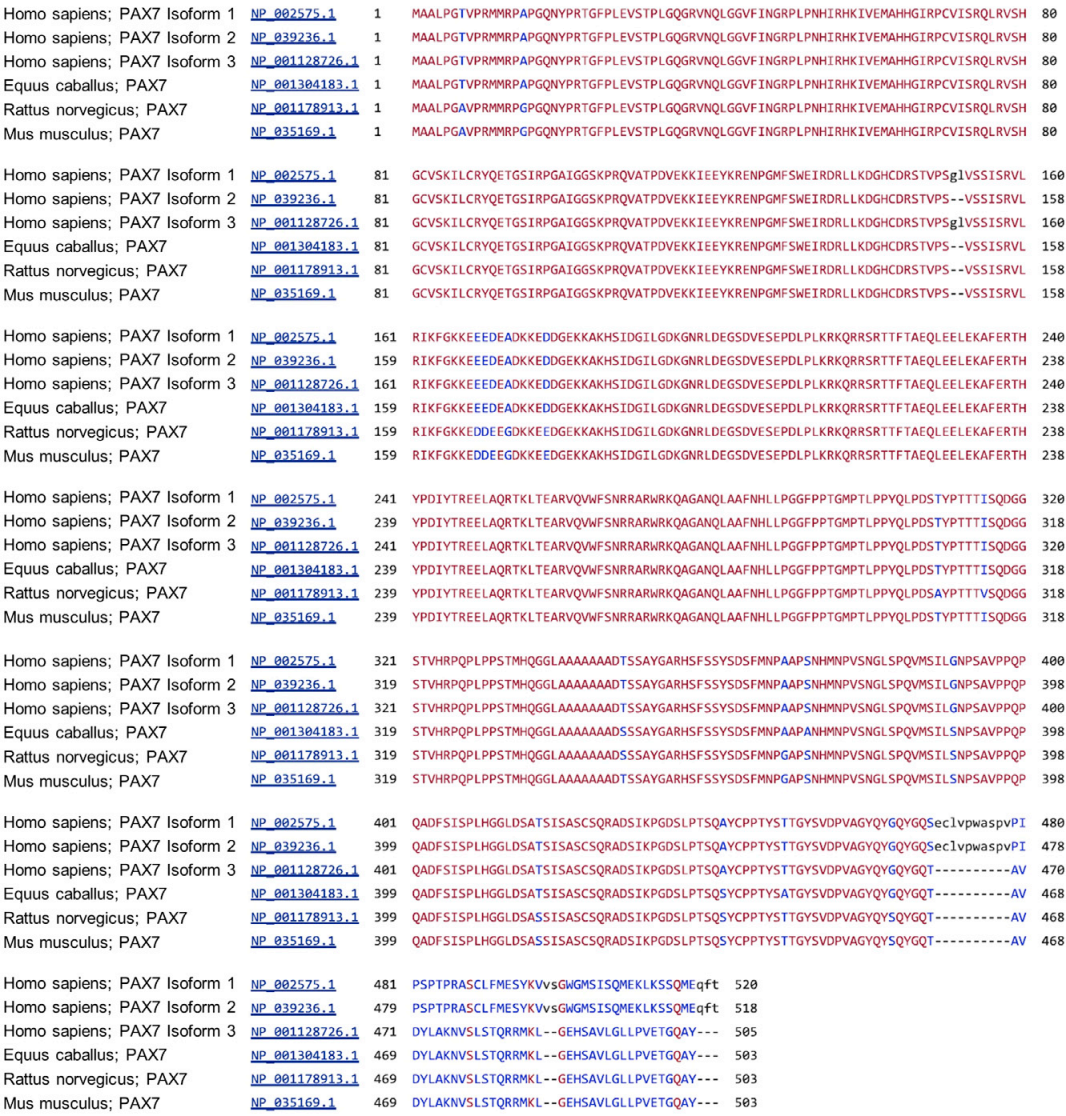
Human PAX7 Protein Isoform Conservation across Mammals The sequences of the three human PAX7 protein isoforms are aligned to verified PAX7 protein sequences (NCBI accession# starting with “NP_”) of selected mammals using the NCBI COBALT tool. Note that the human isoform 3 but not 1 or 2 is conserved with other mammals at the C terminus.

**CRITICAL:** In the NCBI Gene database, the human *PAX7* gene (Gene ID: 5081) has three transcript variants encoding three different protein isoforms: https://www.ncbi.nlm.nih.gov/gene/5081#reference-sequences. Among the three transcript variants, variant 3 (NM_001135254) encodes protein isoform 3 (NP_001128726) which is conserved in mammals (Figure 1). Moreover, it has been reported that transcript variant 3 and the corresponding protein isoform 3 are predominantly expressed in human cells (Barr et al., 1999; Du et al., 2005; Vorobyov and Horst, 2004). Therefore, we chose to insert the reporter cassette in the 3′ untranslated region (UTR) to reflect on the expression of *PAX7* transcript variant 3 (*PAX7.v3*).

***Alternatives:*** If no influence on transcriptional activation can be confirmed, one can insert the reporter in the 5′ UTR of *PAX7*. As the 5′ regions of the three transcript variants are identical, this design will report on overall *PAX7* expression regardless of variants transcribed.

2. Retrieve and select the optimal region of the 3′ UTR to design candidate gRNAs for CRISPR-mediated knockin of the reporter cassette.

***Note:*** To eliminate any potential influences of a fluorescent tag on the endogenous PAX7 protein function, we decided to use an internal ribosomal entry site (IRES) to achieve expression of *PAX7* and the reporter on a single transcript while allowing translation into separate proteins.

**Figure 2:**
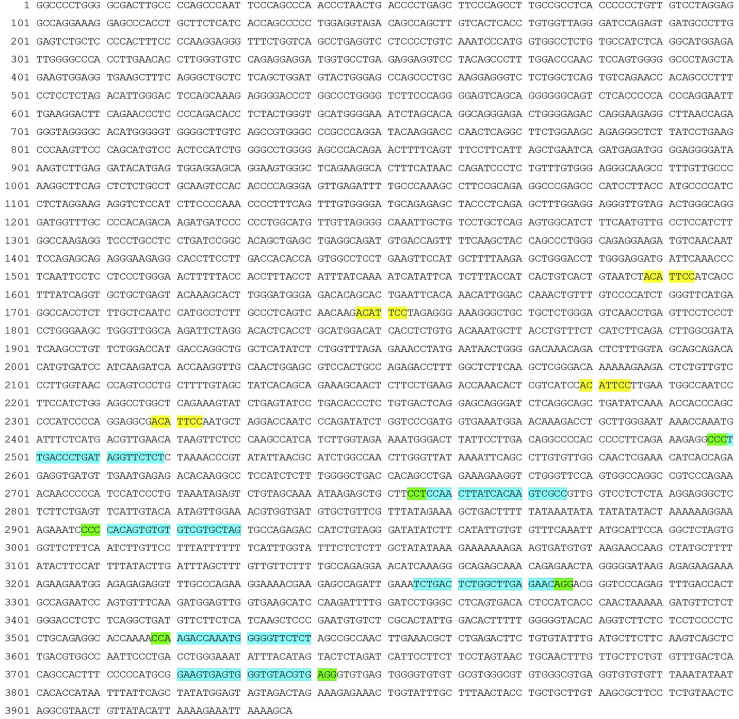
Predicted miRNA Binding Sites and Candidate gRNA Targeting Sequences in *PAX7.v3* 3′ UTR The genomic sequence of the 3′ UTR of *PAX7.v3* is shown. The 7-mer core nucleotides of predicted human mir-1 family (hsa-mir-206/hsa-mir-1-1/hsa-mir-1-2) binding sites are shown in yellow. The 20 bp candidate gRNA sequences followed by the NGG PAM sequences are depicted in turquoise and green, respectively. The 17 bp form of gRNAs are designed to the same regions as their 20 bp counterparts.

***Note:*** The 3′ UTR of *PAX7.v3* contains potential miRNA binding sites of human mir-1 family (hsa-mir-206/hsa-mir-1-1/hsa-mir-1-2) predicted by miRBase (http://www.mirbase.org/) and TargetScan (http://www.targetscan.org/vert_72/) (Figure 2). As the mouse counterparts of these miRNAs have been shown to regulate Pax7 expression (Chen et al., 2010), we limited the gRNA targeting region to the last 1,500 bp of the 3′ UTR that is downstream of these potential miRNA binding sites.

3. Submit the selected 3′ UTR sequence of *PAX7.v3* to the Feng Zhang lab CRISPR gRNA tool (http://www.crispr.mit.edu/) to design candidate gRNAs.

***Note:*** Many gRNA candidates are present within the 1,500 bp selected 3′ UTR. To minimize the risk from unknown genomic structures or epigenomic modifications that might negatively impact CRISPR/Cas9 efficiency, avoid choosing gRNA candidates that are clustered in the same region. In our case, we picked six candidate gRNAs across the entire 1,500 bp sequence (Figure 2).

***Note:*** It has been shown that compared to the regular 20 bp gRNA, the shorter 17 bp form possesses increased target specificity (Fu et al., 2014). Therefore, we also designed 17 bp (short or S) gRNAs based on the six 20 bp (long or L) gRNAs mentioned above, resulting in a total of 12 candidate gRNAs for further testing.

***Note:*** The Feng Zhang lab tool (http://www.crispr.mit.edu/) we used to design gRNAs has been decommissioned as the manuscript was being prepared. However, the new webpage (https://zlab.bio/guide-design-resources) lists a variety of tools that can be used to efficiently design gRNAs.

4. Order DNA oligos for each candidate gRNA from IDT or other companies to be used for constructing gRNA expression plasmids (Table 1).

**Table 1:** Oligos for gRNA Plasmid Construction for Reporter Insertion

Oligo Pair Name	Oligo Sequence	
*PAX7_1-11_L*	Forward	**GGAGAACCTATCAGGGTCAA***GTTTTAGAGCTAGAAATAGCAAGTTAAAATAAGGCTAGTC*
	Reverse	TTGACCCTGATAGGTTCTCC*GGTGTTTCGTCCTTTCCACAAGATATATAAAGCCAAGAAA*
*PAX7_1-11_S*	Forward	**GAACCTATCAGGGTCAA***GTTTTAGAGCTAGAAATAGCAAGTTAAAATAAGGCTAGTC*
	Reverse	TTGACCCTGATAGGTTC*GGTGTTTCGTCCTTTCCACAAGATATATAAAGCCAAGAAA*
*PAX7_2-4_L*	Forward	**GGCGACTTGTGATAAGTTGG***GTTTTAGAGCTAGAAATAGCAAGTTAAAATAAGGCTAGTC*
	Reverse	CCAACTTATCACAAGTCGCC*GGTGTTTCGTCCTTTCCACAAGATATATAAAGCCAAGAAA*
*PAX7_2-4_S*	Forward	**GACTTGTGATAAGTTGG***GTTTTAGAGCTAGAAATAGCAAGTTAAAATAAGGCTAGTC*
	Reverse	CCAACTTATCACAAGTC*GGTGTTTCGTCCTTTCCACAAGATATATAAAGCCAAGAAA*
*PAX7_3-7_L*	Forward	**GTAGCACGACACACACTGTG***GTTTTAGAGCTAGAAATAGCAAGTTAAAATAAGGCTAGTC*
	Reverse	CACAGTGTGTGTCGTGCTAC*GGTGTTTCGTCCTTTCCACAAGATATATAAAGCCAAGAAA*
*PAX7_3-7_S*	Forward	**GCACGACACACACTGTG***GTTTTAGAGCTAGAAATAGCAAGTTAAAATAAGGCTAGTC*
	Reverse	CACAGTGTGTGTCGTGC*GGTGTTTCGTCCTTTCCACAAGATATATAAAGCCAAGAAA*
*PAX7_4-11_L*	Forward	**GCTGACTCTGGCTTGAGAAC***GTTTTAGAGCTAGAAATAGCAAGTTAAAATAAGGCTAGTC*
	Reverse	GTTCTCAAGCCAGAGTCAGC*GGTGTTTCGTCCTTTCCACAAGATATATAAAGCCAAGAAA*
*PAX7_4-11_S*	Forward	**GACTCTGGCTTGAGAAC***GTTTTAGAGCTAGAAATAGCAAGTTAAAATAAGGCTAGTC*
	Reverse	GTTCTCAAGCCAGAGTC*GGTGTTTCGTCCTTTCCACAAGATATATAAAGCCAAGAAA*
*PAX7_5-10_L*	Forward	**GGAGAACCCCCATTTGGTCT***GTTTTAGAGCTAGAAATAGCAAGTTAAAATAAGGCTAGTC*
	Reverse	AGACCAAATGGGGGTTCTCC*GGTGTTTCGTCCTTTCCACAAGATATATAAAGCCAAGAAA*
*PAX7_5-10_S*	Forward	**GAACCCCCATTTGGTCT***GTTTTAGAGCTAGAAATAGCAAGTTAAAATAAGGCTAGTC*
	Reverse	AGACCAAATGGGGGTTC*GGTGTTTCGTCCTTTCCACAAGATATATAAAGCCAAGAAA*
*PAX7_6-9_L*	Forward	**GAAGTGAGTGGGTGTACGTG***GTTTTAGAGCTAGAAATAGCAAGTTAAAATAAGGCTAGTC*
	Reverse	CACGTACACCCACTCACTTC*GGTGTTTCGTCCTTTCCACAAGATATATAAAGCCAAGAAA*
*PAX7_6-9_S*	Forward	**GTGAGTGGGTGTACGTG***GTTTTAGAGCTAGAAATAGCAAGTTAAAATAAGGCTAGTC*
	Reverse	CACGTACACCCACTCAC*GGTGTTTCGTCCTTTCCACAAGATATATAAAGCCAAGAAA*

Bold type indicates spacers; underlining indicates equivalent to protospacers on the genome, with the last nucleotide replaced with a C when it is not; italic type indicates adaptors for Gibson cloning into the backbone vector.

### Design of gRNAs for Reporter Validation

**Timing: 1 day**

5. Retrieve the sequence of human *PAX7* promoter region based on published literature (Murmann et al., 2000).
6. Use the Feng Zhang lab CRISPR gRNA tool (http://www.crispr.mit.edu/) to design gRNAs.

***Note:*** As different promoter targeting gRNAs could result in various activation levels and multiple gRNAs might be required to achieve strong gene expression, we designed four gRNAs spanning the *PAX7* promoter region to be used to activate endogenous *PAX7* expression for reporter validation.

***Note:*** The Feng Zhang lab tool (http://www.crispr.mit.edu/) we used to design gRNAs has been decommissioned as the manuscript was being prepared. However, the new webpage (https://zlab.bio/guide-design-resources) lists a variety of tools that can be used to efficiently design gRNAs.

7. Order DNA oligos for each gRNA from IDT or other companies to be used for constructing gRNA expression plasmids (Table 2).

**Table 2:** Oligos for gRNA Plasmid Construction for Reporter Validation

Oligo Pair Name	Oligo Sequence	
*PAX7_C3*	Forward	GTCAAACGCGTCCAGAAGCTGTTTTAGAGCTAGAAATAGCAAGTTAAAATAAGGCTAGTC
	Reverse	AGCTTCTGGACGCGTTTGACGGTGTTTCGTCCTTTCCACAAGATATATAAAGCCAAGAAA
*PAX7_C4*	Forward	GGGGCCAAAGTTTCCGAGCCGTTTTAGAGCTAGAAATAGCAAGTTAAAATAAGGCTAGTC
	Reverse	GGCTCGGAAACTTTGGCCCCGGTGTTTCGTCCTTTCCACAAGATATATAAAGCCAAGAAA
*PAX7_C5*	Forward	GGGTCCGGAGAAAGAAGGCGGTTTTAGAGCTAGAAATAGCAAGTTAAAATAAGGCTAGTC
	Reverse	CGCCTTCTTTCTCCGGACCCGGTGTTTCGTCCTTTCCACAAGATATATAAAGCCAAGAAA
*PAX7_C6*	Forward	GCCCCGGCTCGACCTCGTTTGTTTTAGAGC TAGAAATAGCAAGTTAAAATAAGGCTAGTC
	Reverse	AAACGAGGTCGAGCCGGGGCGGTGTTTCGTCCTTTCCACAAGATATATAAAGCCAAGAAA

## Key Resources Table

**Table:** 

REAGENT or RESOURCE	SOURCE	IDENTIFIER
**Antibodies**		

Anti-PAX7	DSHB	Cat#: PAX7; RRID: AB_528428
Anti-NANOG	Cell Signaling Technology	Cat# 3580; RRID: AB_2150399
Anti-OCT-4A	Cell Signaling Technology	Cat# 2840; RRID: AB_2167691
Anti-SOX2	Cell Signaling Technology	Cat# 3579; RRID: AB_2195767

**Bacterial and Virus Strains**		

NEB 10-beta Competent *E. coli* (High Efficiency)	New England BioLabs	Cat#: C3019H

**Chemicals, Peptides, and Recombinant Proteins**		

Y-27632	Tocris	Cat#: 1254; CAS#: 129830-38-2
CHIR99021	Tocris	Cat#: 4423; CAS#: 252917-06-9
LDN193189	Tocris	Cat#: 6053; CAS#: 1435934-00-1
SB431542	Tocris	Cat#: 1614; CAS#: 301836-41-9
FGF2/bFGF	Proteintech	Cat#: HZ-1285
Recombinant human HGF (HEK293 derived)	PeproTech	Cat#: 100-39H
LONG R3 IGF-I human	Sigma-Aldrich	Cat#: I1271
mTeSR1	StemCell Technologies	Cat#: 85850
DMEM/F-12, HEPES	Thermo Fisher Scientific	Cat#: 11330032
DMEM, high glucose	Thermo Fisher Scientific	Cat#: 11965092
Insulin-Transferrin-Selenium (ITS -G)	Thermo Fisher Scientific	Cat#: 41400045
Fetal bovine serum (FBS)	Thermo Fisher Scientific	Cat#: 16000044
Knockout serum replacement	Thermo Fisher Scientific	Cat#: 10828028
Chick embryo extract	United States Biological	Cat#: C3999
GlutaMAX	Thermo Fisher Scientific	Cat#: 35050061
Nonessential amino acids (NEAA)	Thermo Fisher Scientific	Cat#: 11140076
Sodium Pyruvate	Thermo Fisher Scientific	Cat#: 11360070
Penicillin-Streptomycin	Thermo Fisher Scientific	Cat#: 15140122
Geneticin Selective Antibiotic (G418 Sulfate)	Thermo Fisher Scientific	Cat#: 10131027
Matrigel hESC-Qualified	Corning	Cat#: 354277
TrypLE Express	Thermo Fisher Scientific	Cat#: 12605010
Collagenase IV	Thermo Fisher Scientific	Cat#: 17104019

**Critical Commercial Assays**		

QIAprep Spin Miniprep Kit	QIAGEN	Cat#: 27106
EndoFree Plasmid Maxi Kit	QIAGEN	Cat#: 12362
DNeasy Blood & Tissue Kit	QIAGEN	Cat#: 69504
DNA Clean & Concentrator-5 Kit	Zymo Research	Cat#: D4013
MinElute Gel Extraction Kit	QIAGEN	Cat#: 28604
Q5 Hot Start High-Fidelity 2× Master Mix	New England BioLabs	Cat#: M0494S
AccuPrime Taq DNA Polymerase, high fidelity	Thermo Fisher Scientific	Cat#: 12346086
AflII	New England BioLabs	Cat#: R0520S
NEBuilder HiFi DNA Assembly Master Mix	New England BioLabs	Cat#: E2621S
TransIT-293 Transfection Reagent	Mirus	Cat#: MIR 2700
P3 Primary Cell 4D-Nucleofector X Kit L	Lonza	Cat#: V4XP-3024
ViaFect Transfection Reagent	Promega	Cat#: E4981
SURVEYOR Mutation Detection Kit for Standard Gel Electrophoresis	Integrated DNA Technologies	Cat#: 706020
RNeasy Plus Micro Kit	QIAGEN	Cat#: 74034
iScript Reverse Transcription Supermix for RT-qPCR	Bio-Rad	Cat#: 1708841
SsoAdvanced Universal SYBR Green Supermix	Bio-Rad	Cat#: 1725274

**Experimental Models: Cell Lines**		

Human: 293FT Cell Line	Thermo Fisher Scientific	Cat#: R70007
Human: H9 (WA09) hESC line	WiCell	RRID: CVCL_9773

**Oligonucleotides**		

Primer pairs for qRT-PCR	Xi et al., 2017	N/A
Recombinant DNA		
gRNA_Cloning Vector	Mali et al., 2013	RRID: Addgene_41824
hCas9	Mali et al., 2013	RRID: Addgene_41815
Oct4-IRES-eGFP-PGK-Neo	Yang et al., 2013	RRID: Addgene_48681
SP-dCas9-VPR	Chavez et al., 2015	RRID: Addgene_63798

**Software and Algorithms**		

Primer3Plus	Untergasser et al., 2007	http://www.bioinformatics.nl/cgi-bin/primer3plus/primer3plus.cgi/
Constraint-based Multiple Alignment Tool (COBALT)	NCBI	https://www.ncbi.nlm.nih.gov/tools/cobalt/re_cobalt.cgi
TargetScanHuman	Agarwal et al., 2015	http://www.targetscan.org/vert_72/

**Other**		

4D-Nucleofector Core Unit	Lonza	AAF-1002B
4D-Nucleofector X Unit	Lonza	AAF-1002X
Cytospin 4 Cytocentrifuge	Thermo Fisher Scientific	Cat#: A78300003
Shandon Double Cytofunnel	Thermo Fisher Scientific	Cat#: 1102547
Superfrost Plus Microscope Slides	Thermo Fisher Scientific	Cat#: 4951PLUS
Falcon 100 μm Cell Strainer	Corning	Cat#: 352360
Falcon 70 μm Cell Strainer	Corning	Cat#: 352350
Falcon 5 mL Round Bottom Polystyrene Test Tube, with Cell Strainer Snap Cap	Corning	Cat#: 352235

## Materials and Equipment

**Table:** 293 Medium

Reagent	Final Concentration	Volume
DMEM, high glucose	n/a	432.5 mL
Fetal bovine serum (FBS)	10%	50 mL
GlutaMAX (100×)	1×	5 mL
Nonessential amino acids (NEAA) (100×)	1×	5 mL
Sodium pyruvate (100 mM)	1 mM	5 mL
Penicillin-Streptomycin (10,000 U/mL) (optional)	50 U/mL	2.5 mL
**Total**	**n/a**	**500 mL**

**Table:** SC Medium

Reagent	Final Concentration	Volume
DMEM, high glucose	n/a	392.45 mL
FBS	20%	100 mL
Chick embryo extract	1%	5 mL
FGF2/bFGF (40 μg/mL)	20 ng/mL	50 μL
Penicillin-Streptomycin (10,000 U/mL) (optional)	50 U/mL	2.5 mL
**Total**	**n/a**	**500 mL**

**Table:** hPSC Culture Medium

Reagent	Final Concentration	Volume
mTeSR1 basal medium	n/a	397.5 mL
mTeSR1 supplement (5×)	1×	100 mL
Penicillin-Streptomycin (10,000 U/mL) (optional)	50 U/mL	2.5 mL
**Total**	**n/a**	**500 mL**

***Note:*** Prepare the complete hPSC culture medium and store at 4°C and use within 2 weeks. Smaller aliquots (e.g., 50 mL) can be prepared and stored at −20°C for at least a few months. Once the frozen aliquots are thawed, mix thoroughly to dissolve any precipitated salts and use within 1–2 weeks. Do not refreeze.

**Table:** hPSC Selection Medium

Reagent	Final Concentration	Volume
hPSC culture medium	n/a	499 mL
Geneticin (G418/neomycin) (50 mg/mL)	100 μg/mL	1 mL
**Total**	**n/a**	**500 mL**

**Table:** Basal Differentiation Medium (BDM)

Reagent	Final Concentration	Volume
DMEM/F12, HEPES	n/a	492.5 mL
Insulin-Transferrin-Selenium (100×)	1×	5 mL
Penicillin-Streptomycin (10,000 U/mL) (optional)	50 U/mL	2.5 mL
**Total**	**n/a**	**500 mL**

**Table:** Presomitic Mesoderm (PSM) Promoting Medium

Reagent	Final Concentration	Volume
BDM	n/a	99.985 mL
CHIR99021 (20 mM)	3 μM	15 μL
**Total**	**n/a**	**100 mL**

***Note:*** Take only a sufficient amount of the basal differentiation medium (BDM) for the day. Add the CHIR99021 stock solution to BDM to reach the desired final concentration and use the supplemented medium the same day. Do not store for more than one day since small molecules that dissolve in DMSO are not stable in aqueous solutions. This also applies to the somite (SM) and dermomyotome (DM) promoting medium listed below.

**Table:** Somite (SM) Promoting Medium

Reagent	Final Concentration	Volume
BDM	n/a	99.96 mL
LDN193189 (1 mM)	200 nM	20 μL
SB431542 (50 mM)	10 μM	20 μL
**Total**	**n/a**	**100 mL**

**Table:** Dermomyotome (DM) Promoting Medium

Reagent	Final Concentration	Volume
BDM	n/a	99.9 mL
CHIR99021 (20 mM)	10 μM	50 μL
FGF2/bFGF (40 μg/mL)	20 ng/mL	50 μL
**Total**	**n/a**	**100 mL**

**Table:** Myogenesis (Myo) Promoting Medium

Reagent	Final Concentration	Volume
DMEM, high glucose	n/a	421.95 mL
Knockout serum replacement	15%	75 mL
Recombinant human HGF (HEK293 derived) (10 μg/mL)	10 ng/mL	500 μL
LONG R^3^ IGF-I human (20 μg/mL)	2 ng/mL	50 μL
Penicillin-Streptomycin (10,000 U/mL) (optional)	50 U/mL	2.5 mL
**Total**	**n/a**	**500 mL**

***Note:*** Store the complete myogenesis (Myo) promoting medium at 4°C and use within 2–4 weeks.

## Step-By-Step Method Details

### Cloning of Candidate gRNA Expression Plasmids for Reporter Insertion

**Timing: 1–2 weeks**

This step constructs plasmids expressing candidate gRNAs to be tested for CRISPR/Cas9-mediated DNA cutting efficiencies.

1. Add 1 μg of the gRNA cloning vector (Addgene, #41824) along with 1 μL (20 units) of AflII restriction enzyme to a total of 50 μL reaction volume supplemented with H_2_O. Incubate at 37°C for 1 h.
2. Purify the digested vector using the DNA Clean & Concentrator-5 Kit according to manufacturer’s instructions.

***Optional:*** If not immediately subjected to DNA purification or gel electrophoresis, the digested product should be heat-inactivated at 65°C for 20 min before storage.

3. Ligate the linearized vector with each pair of forward and reverse DNA oligos containing each candidate gRNA targeting site using the NEBuilder HiFi DNA Assembly Master Mix. Prepare the reactions as in the following table. Incubate at 50°C for 15 min and then keep on ice.

**Table:** 

Components	Amount
Digested vector	0.015 pmol
Forward oligo	0.075 pmol
Reverse oligo	0.075 pmol
NEBuilder HiFi DNA Assembly Master Mix	10 μL
H_2_O	Supplement to 20 μL
Total	20 μL

***Note:*** Due to our design, each pair of the forward and reverse DNA oligos partially overlap and leave 3′ overhangs on both ends, which are compatible to anneal to the digested and 5′-chewed vector backbone in the NEBuilder HiFi DNA Assembly reactions. Therefore, it is not necessary to pre-anneal the oligos in separate reactions before ligation.

4. Use 2 μL of the assembled product to transform the NEB 10-beta competent cells following manufacturer’s instructions. Spread different dilutions of the transformed cells onto kanamycin selective LB agar plates and incubate them at 37°Cfor ~16 h.
5. Early the next day, stop incubation when good-sized colonies have formed (in general takes ~16 h). Seal and keep the plates on benchtop.
6. Later the same day, inoculate 1–3 individual colonies per cloned candidate gRNA expression plasmid into 3–5 mL of kanamycin selective LB medium. Grow in a shaker at 37°Cfor ~16 h. Seal the agar plates tightly and store them at 4°C as backups in case more colonies need to be picked.

***Note:*** The 5′ → 3′ exonuclease in the NEBuilder HiFi DNA Assembly Master Mix will destroy the cleaved AflII restriction site, thus preventing vector self-ligation. Include an additional reaction with digested vector only to monitor any false positive events.

7. On the following day, stop growing the bacteria when the liquid culture is dense and healthy (~16 h). Take most of the culture to extract plasmid DNA using the QIAprep Spin Miniprep Kit according to manufacturer’s instructions. Mix the remaining liquid culture with an equal volume of 50% glycerol and store long-term at −80°C.
8. Submit the extracted plasmids for Sanger sequencing to verify successful cloning of the candidate gRNAs using the T7 sequencing primer (Table 3).

**Table 3:** Sanger Sequencing Primers

Primer Name	Primer Sequence
*Seq primer AmpR*	CAGATTACGCGCAGAAAA
*Seq primer Backbone 5′*	CCTGTGCTATGGAGAATTCCTG
*Seq primer Backbone 3′*	GCTTATCGATACCGTCGACCTC
*Seq primer BGHR*	TAGAAGGCACAGTCGAGG
*Seq primer EGFP*	TACCGGTGGATGTGGAAT
*Seq primer EGFP-C*	CATGGTCCTGCTGGAGTTCGTG
*Seq primer EGFP-N*	CGTCGCCGTCCAGCTCGACCA
*Seq primer external forward #2*	TGGTCCCGATGGTGAAATGG
*Seq primer external reverse #2*	GTTTTAGGGCCAGGGGAACA
*Seq primer internal forward*	AATAAGGCCGGTGTGCGTTT
*Seq primer internal reverse #2*	AACAGACCTTGCATTCCTTT
*Seq primer M13R*	CAGGAAACAGCTATGAC
*Seq primer M13(-47)*	GTTTTCCCAGTCACGAC
*Seq primer Neo-F*	CGTTGGCTACCCGTGATATT
*Seq primer Neo-R*	TGGATACTTTCTCGGCAG
*Seq primer PAX7 L-HA*	AGAACCAAGCTATGCTTTTATACTTCC
*Seq primer PAX7 R-HA*	GTGTACGTGAGGGTGTGAGTG
*Seq primer PAX7 R-HA-R*	ATGATCACACCTGTGAATAGCCTTG
*Seq primer T7*	TAATACGACTCACTATAGGG

**Pause Point:** Keep the glycerol stocks harboring the correctly cloned plasmids and discard those that are false positives or with incorrect insertions. At this point, the glycerol stocks at −80°C can be stored for long-term and you can proceed to the next steps at a later time.

9. Thaw the glycerol stocks on ice. Streak kanamycin selective LB agar plates and incubate them at 37°Cfor ~16 h.
10. Early the next day (after ~16 h of incubation), inoculate a single colony from each plate into 3–5 mL of kanamycin selective LB medium. Grow bacteria for ~8 h at 37°C in a shaker, followed by taking a small volume and add into fresh 100 mL of kanamycin selective LB medium at a 1:500 dilution. Grow bacteria at 37°C in a shakerfor ~16 h.
11. On the following day (after ~16 h of culture), use the EndoFree Plasmid MaxiKit to extract transfection-grade plasmids according to manufacturer’s instructions. Store the plasmids at 4°C for short-term or −20°C for long-term.

### Initial Screening of Candidate gRNAs in 293FT Cells

**Timing: 1–2 weeks**

This initial screening step tests the cutting efficiencies of the candidate gRNAs in an easy-to-transfect cell type (here we use 293FT cells). It excludes gRNAs that show no or low cutting efficiencies in 293FT cells, which are unlikely to work in hard-to-transfect hPSCs.

12. Maintain 293FT cells in 293 medium in a humidified 37°C incubator with 5% CO_2_ and change medium every other day. Passage cells before they reach over 70%–80% confluent to avoid overgrowth for optimal transfection performance.
13. One day before transfection (day −1), seed cells at 75,000/cm^2^ onto 24-well tissue culture plates.
14. The next day (day 0), transfect 293FT cells using the TransIT-293 reagent according to manufacturer’s instructions. We transfect a total of 1 μg of plasmids with 0.5 μg each of Cas9 (Addgene, #41815) and gRNA expression plasmid per well on a 24-well plate with a reagent-to-plasmid ratio of 3:1.
15. On day 1, feed cells with fresh medium.
16. On day 3, harvest cells and extract genomic DNA using the DNeasy Blood & Tissue Kit following manufacturer’s instructions.

**Pause Point:** Extracted genomic DNA can be stored long-term at −20°C for later analysis.

17. Use the Q5 Hot Start High-Fidelity 2× Master Mix to PCR amplify the genomic sequence encompassing the cut site of each candidate gRNA. Assembly of the PCR reactions and the cycling conditions are as follows (see Table 4 for primer sequences).

**Table:** 

Components	Amount
Template genomic DNA	250 ng
Forward primer	500 nM
Reverse primer	500 nM
Q5 High-Fidelity 2× Master Mix	12.5 μL
H_2_O	Supplement to 25 μL
Total	25 μL

PCR Cycling Conditions			
Steps	Temperature	Time	Cycles
Initial denaturation	98°C	30 s	1
Denaturation	98°C	10 s	30–35
Annealing	60°C–62°C^a^	20 s	
Extension	72°C	30 s	
Final extension	72°C	2 min	1
Hold	4°C	Forever	

a Set annealing temperature at 62°C for PAX7 Surveyor primer pairs 1–11, 4–11, 5–10 and 6–9, and 60°C for 2–4 and 3–7.

**Table 4:** Surveyor Assay PCR Primers

Primer Pair Name	Primer Sequence	
*Surveyor PAX7_1-11*	Forward	TCCACTGCCAGAGACCTTTG
	Reverse	CAGCACATCACCACGTTTCC
*Surveyor PAX7_2-4*	Forward	GAGGCCCTTGACCCTGATAG
	Reverse	CGAGACACATTCGGGAGCTT
*Surveyor PAX7_3-7*	Forward	TATCAAAACCACCCAGCCCC
	Reverse	CGTCCTGTTCTCAAGCCAGA
*Surveyor PAX7_4-11*	Forward	GGAAAGAAATCCCCCACAGT
	Reverse	CTCACACCCTCACGTACACC
*Surveyor PAX7_5-10*	Forward	GAGGAACATCAAAGGGCAGA
	Reverse	CAGAGTCCCTTTTGGAGCAG
*Surveyor PAX7_6-9*	Forward	GAGGAACATCAAAGGGCAGA
	Reverse	AGTGGGGAGCAAGAGAAACA

**CRITICAL:** The efficient design of PCR primers flanking the cut site of each candidate gRNA is important to the successful detection of cutting events in the downstream Surveyor assay. PCR amplicons that are too short will be difficult to analyze using standard DNA gel electrophoresis. On the other hand, PCR products that are too long will be hard to amplify and are more susceptible to potential PCR-introduced mutations that could result in false positives in the Surveyor assay. Moreover, it is ideal to design the PCR products so that two fragments with different sizes are produced after Surveyor nuclease digestion to facilitate downstream detection. We were able to design primers that produced amplicons all around 1 kb to balance the above considerations. However, the primer design for your own experiments will be influenced by the cutting sites of choice and the feasibility to design good primers and amplicons from the surrounding sequences.

18. Run 5 μL of the PCR products on a 2% TAE agarose gel to estimate the PCR product concentrations and ensure specific amplification (no unspecific bands near the sizes of expected fragments to be generated in the downstream Surveyor assay).

19. Perform the Surveyor assay.
  a. Add 200–300 ng of the remaining PCR products into separate PCR tubes and supplement with H_2_O to a final volume of 10–20 μL. Run the following denature and gradual re-annealing program in a thermal cycler to form DNA heteroduplex.

**Table:** 

Temperature	Time	Temperature ramp
95°C	10 min	
95°C to 85°C		−2.0°C/s
85°C	1 min	
85°C to 75°C		−0.3°C/s
75°C	1 min	
75°C to 65°C		−0.3°C/s
65°C	1 min	
65°C to 55°C		−0.3°C/s
55°C	1 min	
55°C to 45°C		−0.3°C/s
45°C	1 min	
45°C to 35°C		−0.3°C/s
35°C	1 min	
35°C to 25°C		−0.3°C/s
25°C	1 min	
4°C	forever
  b. After hybridization, add the following components from the Surveyor Mutation Detection Kit for Standard Gel Electrophoresis and incubate at 42°C for 60 min to digest the mismatched DNA double strands.

**Table:** 

Component	Amount
Hybridized DNA	10–20 μL
MgCl_2_ Solution (0.15 M)	10% of final total volume
Surveyor Enhancer S	1 μL
Surveyor Nuclease S	2 μL

    ***Note:*** The final Mg^2+^ concentration and Surveyor Nuclease S amount for optimal DNA heteroduplex digestion are dependent on the buffer conditions of the PCR reactions. Refer to the Surveyor assay user guidelines for adjustment of these components according to your choice of PCR reagents.
  c. Stop digestion by adding 10% reaction volume of the Stop Solution.
20. Run the entire digested products on a 2% TAE agarose gel. Pick the candidate gRNAs that show clear bands with correct sizes of expected digested PCR fragments (reflecting successful CRISPR/Cas9 mediated genomic DNA cutting) and quantify the cutting efficiencies (Figure 3A). Use the following formula (Guschin et al., 2010) for estimating CRISPR/Cas9-introduced small insertions and deletions (indels): indel% = (1-(1-(B+C)/(A+B+C))ˆ0.5)∗100, where A represents the integrated band intensity of the uncleaved PCR product and B and C of the two cleaved products.

**Figure 3:**
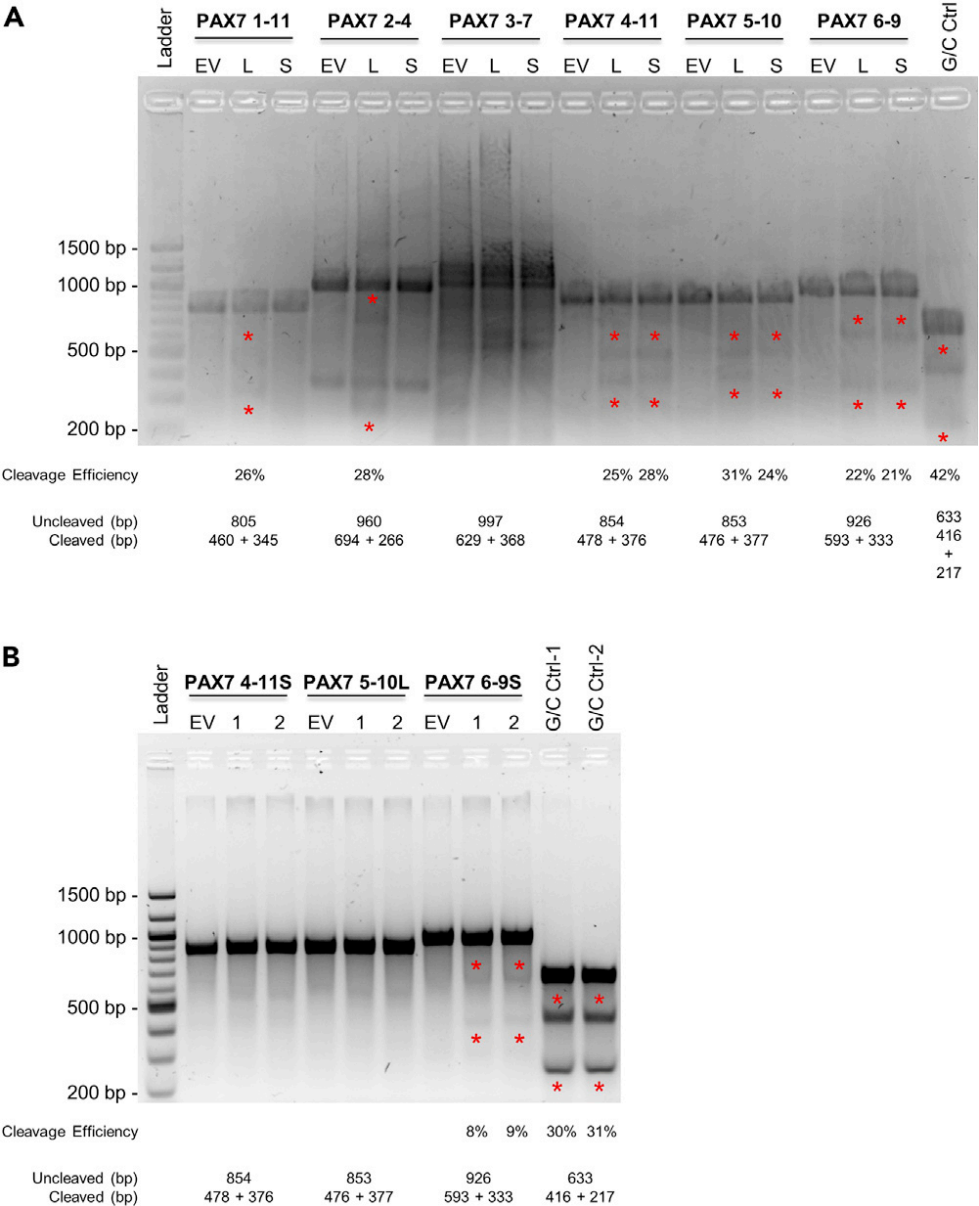
Gel Electrophoresis of Surveyor Assay Products from 293FT or H9 Cells Transfected with Different Candidate gRNAs (A) 293FT cells were co-transfected with plasmids encoding Cas9 and one of the 20 bp (L) or 17 bp (S) gRNAs. An additional transfection was performed with the Cas9 plasmid and empty gRNA cloning vector (EV). Genomic sequences surrounding candidate gRNA cutting sites were PCR amplified using indicated primer pairs and subsequently subjected to Surveyor assay digestion. Digested fragments corresponding to expected sizes are denoted by “∗” above or below the respective bands. The positive G/C Control included in the assay kit was analyzed along with the other samples. (B) H9 cells were nucleofected with plasmids encoding Cas9 and the gRNAs selected from screening in 293FT cells. Nucleofection for each candidate gRNA was performed in duplicates and processed separately in the downstream Surveyor assay. Fragments and controls are denoted similarly as in (A).

***Note:*** It is recommended to use the G/C Control included in the Surveyor assay kit as a positive control to ensure proper conditions and compatibility of PCR, hybridization, and digestion reactions.

**CRITICAL:** When transfecting cells with candidate gRNA expression plasmids, also include a condition where cells are transfected by the empty gRNA cloning plasmid. Process this condition the same as others for genomic DNA extraction, PCR amplification, and Surveyor assay analysis. This can serve as a control for any potential false positives related to unwanted Cas9 cutting, unspecific PCR amplification, as well as background nuclease digestion introduced, e.g., by SNPs between alleles.

***Alternatives:*** More computationally based methods such as TIDE (Tracking of Indels by DEcomposition) (Brinkman et al., 2014) could also be used in place of enzyme-based assays to estimate CRISPR/Cas9 genome editing efficiencies for candidate gRNAs.

### Secondary Screening of Candidate gRNAs in hPSCs

**Timing: 1–2 weeks**

After initial screening of candidate gRNAs in 293FT cells (or any other easy-to-transfect human cell lines), we picked the most efficient gRNAs PAX7 4-11S, 5-10L and 6-9S to further examine their efficiencies in H9 hPSCs (or other desired hPSC lines for reporter generation).

21. Maintain H9 cells as colonies on Matrigel-coated tissue culture plates using hPSC culture medium in a humidified 37°C incubator with 5% CO_2_ and change medium every day. Passage cells when they reach ~70%–80% confluent using 0.5 mM EDTA solution with 4–5 min incubation in the hood.
22. On the day of nucleofection (day 0), remove spent hPSC culture medium and add fresh medium supplemented with 10 μM of the ROCK inhibitor Y-27632. Incubate the cells at 37°C for 1 h to increase cell viability during the transfection procedures.

***Note:*** Use cells that are no more than 50% confluent or colonies not too compact or large. This ensures the majority of cells are in a rapid proliferation state to maximize the transfection efficiency. For H9, EDTA-passaged colonies after ~3 days of culture are ideal.

23. Dissociate into single cells using TrypLE Express with 7–8 min incubation at 37°C.
24. Add at least 3 volumes of hPSC culture medium to TrypLE Express to stop dissociation and transfer the cell solution to a 15 mL conical tube to prevent cells from reattaching to the tissue culture plates.
25. Count and aliquot 800,000 cells into one 1.5 mL Eppendorf tube per nucleofection reaction.
26. Spin down cells, remove supernatant and resuspend cells using 100 μL reconstituted nucleofection solution per reaction prepared by mixing 82 μL Nucleofector Solution and 18 μL of Supplement from the P3 Primary Cell 4D-Nucleofector X Kit L.
27. Add to the cells 2 μg each of the Cas9 and gRNA expression plasmid per reaction.

***Optional:*** Include a separate reaction with the pmaxGFP plasmid accompanied with the kit to monitor nucleofection efficiencies.

**CRITICAL:** Include a separate reaction with the Cas9 and empty gRNA cloning plasmid. This condition can serve as a control for unwanted Cas9 cutting, non-specific PCR amplification, as well as background nuclease digestion in downstream analysis.

28. Transfer the cell/plasmid mixture to the kit-supplied Nucleocuvette Vessels and run program CA-137 on a 4D-Nucleofector X Unit assembled with a Core Unit.

**Figure 4:**
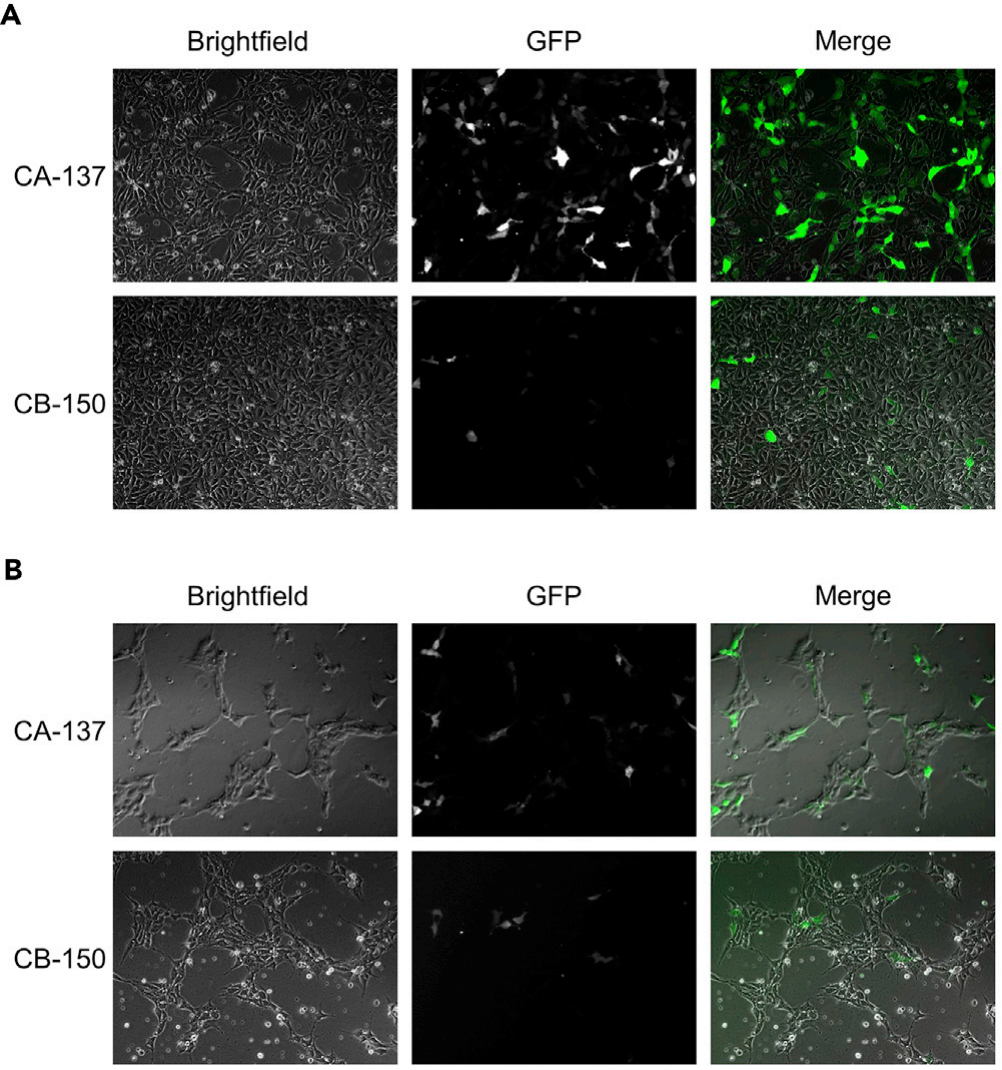
Comparison of Nucleofection Efficiencies of Different Programs (A) H9 cells were nucleofected with the pmaxGFP plasmid using either the CA-137 or CB-150 program and examined for GFP expression 1 day later. (B) An in-house generated iPSC line (CDMD 1006) was nucleofected and examined similarly as described in (A).

***Note:*** The manufacturer recommends program CB-150 for nucleofection of hPSCs including H9 cells. However, in our hands, program CA-137 clearly outperforms CB-150 in H9 as well as other hPSC lines on nucleofection efficiencies (Figure 4). The variation could result from but not limited to the hPSC lines and the specific culture conditions used. Therefore, we highly recommend users to test a few different nucleofection programs for their lines of interest to achieve optimal viability and efficiency.

29. To each Nucleocuvette Vessel, add 0.5 mL of hPSC culture medium supplemented with 10 μM of Y-27632 and transfer the nucleofected cells to 1 well on a 24-well tissue culture plate pre-coated with Matrigel.

**CRITICAL:** Use the kit-supplied transfer pipettes instead of regular pipette tips to add medium and mix and transfer the nucleofected cells from the Nucleocuvette Vessels to the tissue culture wells. Additionally, limit pipetting and mixing nucleofected cells to no more than 2–3 times to increase cell viability.

30. Add another 0.5 mL of hPSC culture medium supplemented with 10 μM of Y-27632 to the well. Shake the plate several times in a back-and-forth and left-and-right motion and culture the cellsfor ~24 h.
31. The next day (day 1), dissociate the cells in each well on the 24-well plate using TrypLE Express and seed them into 1 well on a Matrigel-coated 6-well tissue culture plate. Culture the cells in hPSC culture medium supplemented with 10 μM of Y-27632. Troubleshooting 1
32. On day 2, refresh cells with regular hPSC culture medium without Y-27632.
33. On day 3, harvest cells and extract genomic DNA using the DNeasy Blood & Tissue Kit following manufacturer’s instructions.

**Pause Point:** Extracted genomic DNA can be stored long-term at −20°C for later analysis.

34. Perform downstream analysis of PCR amplification, Surveyor assay and gel electrophoresis (Figure 3B) as described in steps 17–20 in section “Initial Screening of Candidate gRNAs in 293FT Cells.”

### Cloning of Reporter Donor Plasmid

**Timing: 1–2 weeks**

After gRNA screening in 293FT and H9 cells, we decided to use the gRNA 6-9S to mediate Cas9 cutting and insertion of a reporter cassette in the endogenous *PAX7* gene 3′ UTR through homology-directed recombination (HDR). In this step, we describe the procedures to construct the donor plasmid by replacing the *Oct4* targeting homology arms (HAs) from the Oct4-IRES-eGFP-PGK-Neo plasmid (Addgene, #48681) with *PAX7* genomic sequences surrounding the gRNA 6-9S targeted region.

35. Extract genomic DNA from H9 cells and use as template to PCR amplify the left and right HAs (L-HA/650 bp and R-HA/700 bp, respectively; Table 5) for HDR at the *PAX7* locus. Assemble the PCR reactions and run the cycling program as follows.

**Table 5:** Homology-Directed Recombination Donor Vector Cloning PCR Primers

Primer Pair Name	Primer Sequence	
*HDR cloning PAX7 L-HA*	Forward	AGAACCAAGCTATGCTTTTATACTTCC
	Reverse	CCACTCACTTCCGCATGG
*HDR cloning PAX7 R-HA*	Forward	GTGTACGTGAGGGTGTGAGTG
	Reverse	ATGATCACACCTGTGAATAGCCTTG
*HDR cloning Backbone*	Forward	CTATTCACAGGTGTGATCATCCTGTGCTATGGAGAATTCCTG
	Reverse	TAAAAGCATAGCTTGGTTCTGCTTATCGATACCGTCGACCTC
*HDR cloning Reporter*	Forward	CCCCATGCGGAAGTGAGTGGGCTCAGTGATGCTGTTGATCC
	Reverse	ACTCACACCCTCACGTACACTGCAGAATTCGGCTTGTACTG

**Table:** 

Components	Amount
Template genomic DNA	100 ng
Forward primer	500 nM
Reverse primer	500 nM
Q5 High-Fidelity 2× Master Mix	25 μL
H_2_O	Supplement to 50 μL
Total	50 μL

PCR Cycling Conditions			
Steps	Temperature	Time	Cycles
Initial denaturation	98°C	30 s	1
Denaturation	98°C	10 s	30–35
Annealing	66°C	20 s	
Extension	72°C	30 s	
Final extension	72°C	2 min	1
Hold	4°C	Forever

***Note:*** In principle, the HAs can be PCR amplified from genomic DNA extracted from any human source or synthesized through commercial services using reference human genomes. However, subtle genomic differences could exist in distinct cell lines and differ from reference human genomes, which might affect HDR efficiencies. Therefore, we recommend using genomic DNA isolated from the cell lines to be used for reporter generation as the template to prepare HAs.

36. PCR amplify the backbone and reporter cassette (IRES-eGFP-PGK-Neo) from the plasmid. Include 20 bp overlapping sequences with the HAs on the 5′ end of each primer (Table 5) for compatibility in downstream plasmid construction. Assemble the PCR reactions and run the cycling program as follows.

**Table:** 

Components	Amount
Template plasmid	1 ng
Forward primer	500 nM
Reverse primer	500 nM
Q5 High-Fidelity 2× Master Mix	25 μL
H_2_O	Supplement to 50 μL
Total	50 μL

PCR Cycling Conditions			
Steps	Temperature	Time	Cycles
Initial denaturation	98°C	30 s	1
Denaturation	98°C	10 s	25–30
Annealing	66°C	20 s	
Extension	72°C	90 s	
Final extension	72°C	2 min	1
Hold	4°C	Forever

37. Run the entire PCR products from the above two steps on a 1.5% TAE agarose gel, excise the correct-sized bands, and extract DNA using the MinElute Gel Extraction Kit according to manufacturer’s instructions.

***Alternatives:*** Run gel electrophoresis of a small portion of the PCR products. If no overt unspecific amplification is observed, clean the remaining PCR reactions using the DNA Clean & Concentrator-5 Kit according to manufacturer’s instructions.

38. Submit the purified PCR products for Sanger sequencing to exclude any PCR-introduced mutations. Refer to the following table for sequencing primer(s) for each PCR amplicons (see Table 3 for primer sequences).

**Table:** 

PCR Amplicon	Sequencing Primer
*PAX7* L-HA	Seq primer *PAX7* L-HA
*PAX7* R-HA	Seq primer *PAX7* R-HA
Backbone	Seq primer AmpR
	Seq primer Backbone 5′
	Seq primer Backbone 3′
Reporter cassette	Seq primer EGFP
	Seq primer EGFP-N
	Seq primer Neo-F
	Seq primer Neo-R
	Seq primer BGHR

39. Ligate all four PCR fragments using the NEBuilder HiFi DNA Assembly Master Mix. Prepare the reaction as in the following table. Incubate at 50°C for 1 h and then keep on ice.

**Table:** 

Components	Amount
*PAX7* L-HA PCR amplicon	0.04 pmol
*PAX7* R-HA PCR amplicon	0.04 pmol
Backbone PCR amplicon	0.04 pmol
Reporter cassette PCR amplicon	0.04 pmol
NEBuilder HiFi DNA Assembly Master Mix	10 μL
H_2_O	Supplement to 20 μL
Total	20 μL

40. Transform competent cells, grow bacteria and extract plasmids as described in steps 4–7 in section “Cloning of Candidate gRNA Expression Plasmids for Reporter Insertion.” Replace kanamycin with ampicillin in LB agar and medium.
41. Submit the extracted plasmids for Sanger sequencing to verify successful cloning. Use the following sequencing primers to cover the junctional regions of the four PCR fragments (see Table 3 for primer sequences).

**Table:** 

Junctional Region	Sequencing Primer
Backbone - *PAX7* L-HA	Seq primer M13R
*PAX7* L-HA - Reporter cassette	Seq primer EGFP-N
Reporter cassette - *PAX7* R-HA	Seq primer Neo-F
*PAX7* R-HA - Backbone	Seq primer M13(-47)

42. Prepare transfection-grade plasmids as described in steps 9–11 in section “Cloning of Candidate gRNA Expression Plasmids for Reporter Insertion.” Replace kanamycin with ampicillin in LB agar and medium.

### Establishment of Reporter Cell Lines with Antibiotic Resistance

**Timing: 4–6 weeks**

In this step, we describe the procedures of reporter knockin by co-nucleofection of plasmids encoding Cas9, gRNA, and the donor template into H9 hPSCs, followed by establishing single-cell clones post antibiotic selection. We also provide materials and methods for genotyping to check correctly integrated clones.

43. Perform H9 nucleofection as described in steps 21–30 in section “Secondary Screening of Candidate gRNAs in hPSCs” with 2 μg each of the Cas9, *PAX7* 6-9S gRNA, and *PAX7* HDR donor plasmid. Culture the cells for ~24 h with hPSC culture medium supplemented with 10 μM of Y-27632.

***Optional:*** Include a separate reaction with the same amount of the Cas9 and *PAX7* 6-9S gRNA plasmid while replacing *PAX7* HDR donor with the kit-accompanied pmaxGFP plasmid. This can be used to monitor the efficiencies of nucleofection as well as downstream antibiotic selection.

44. The next day, dissociate the cells in 1 well on a 24-well plate using TrypLE Express and seed them into 1 well on a 6-well plate. Culture the cells in hPSC culture medium supplemented with 10 μM of Y-27632.
45. On day 2 post nucleofection, refresh cells with regular hPSC culture medium without Y-27632 and allow the HDR donor-integrated cells to express the antibiotic-resistant protein.
46. On day 3 post nucleofection, start antibiotic selection by feeding cells with G418 (neomycin; 100 μg/mL) hPSC selection medium.

**CRITICAL:** Since different hPSC lines can possess distinct sensitivities to a certain antibiotic, it is important to perform kill curve experiments in advance to establish the optimal antibiotic concentrations for the cell lines of interest. As variables such as cell density, colony compactness, and transfection-related cellular stress can also influence antibiotic sensitivity, we highly recommend processing the cells in the kill curve experiments as similarly as possible to the actual selection process. Accordingly, we established the G418 kill curve in H9 cells 3 days after mock-nucleofection and observed that a concentration of 100 μg/mL was needed to kill all cells over a period of ~5 days.

47. For the next 4–5 days after starting antibiotic selection, you will see massive cell death with only a few single cells or tiny cell clusters (2–3 cells per cluster) remaining in the well. This is normal. Do not discard the cells and keep refreshing them with hPSC selection medium on a daily basis.
48. After ~1 week of antibiotic selection, small resistant colonies start to emerge, and cells grow more rapidly than the initial period of selection.

***Note:*** If the Cas9 + gRNA + pmaxGFP reaction is included for nucleofection, cells subjected to this condition should be all dead at this point.

49. Keep feeding cells with hPSC selection medium. After 1–2 weeks of selection manually detach individual middle-sized colonies (~200–400 cells per colony) with P200 pipette tips and transfer each colony into 1 well on a 24-well plate pre-coated with Matrigel. Change tips and wash the wells after each colony picking to prevent cross-contamination.

**CRITICAL:** Closely monitor the culture during this period of time, and only pick and transfer colonies emerging from single cells. Do not include colonies that you suspect to have already merged together. As the growth rate of individual colonies varies, you will most likely need to stagger the process and pick colonies on different days once suitable ones emerge.

***Note:*** Based on the fact that almost all cells were killed by antibiotic selection under our experimental setting, it is extremely unlikely that 2 or more close-by cells simultaneously integrated the donor plasmid and became resistant. Therefore, it is reasonable to assume that the individual colonies emerged after selection are of single-cell origin. However, if antibiotic selection is not available or there are many cells surviving the selection, it will be impossible to perform single colony picking as described here. In such cases, cell sorting, low density plating, or serial dilution will be required to ensure clonal cell line establishment.

50. For colonies transferred to 24-well plates, let them attach to the culture wells and start feeding daily with hPSC selection medium 2 days post transfer. At the same time, feed the remaining cells in the 6-well plate with hPSC selection medium on a daily basis.

***Note:*** Not all colonies transferred to 24-well plates are suitable for downstream processing. Some colonies might not reattach to the culture wells after transfer. Some colonies might grow a lot slower or faster than normal, which could indicate potential genomic abnormalities and should be discarded. In addition, colonies that show overt spontaneous differentiation should be excluded from downstream processing.

51. During the next week (~week 2–3 after selection starts), use 0.5 mM EDTA to split the colonies in each 24-well to one Matrigel-coated 12-well once they become big in size. Tweak the optimal EDTA incubation time depending on the size and compactness of colonies in each well.

**CRITICAL:** It is important to let the colonies reach a big size (comparable to that of regularly maintained hPSC colonies with at least a few thousand cells) before splitting to allow expansion of the clones. However, avoid keeping growing the colonies without passage until they show signs of overt spontaneous differentiation such as rugged colony edges or brown colony centers. Colonies with slight spontaneous differentiation will most likely recover after 1–2 regular passages.

***Alternatives:*** Colonies can also be split by TrypLE Express dissociation into single cells or manually cutting into smaller pieces and detach and transfer to new culture vessels.

52. During the same period, split the remaining cells in the 6-well to one 12-well. Culture the cells with hPSC selection medium. These pooled “polyclonal” cells can be saved as backups in case more single clones are needed in the future.
53. During the following week (~week 3–4 after selection starts), use 0.5 mM EDTA to split the colonies in each 12-well to multiple 6-wells once they become 70%–80% confluent. Feed cells daily with hPSC selection medium.
54. Once cells reach 70%–80% confluent in 6-wells, freeze them at one 6-well per cryotube using hPSC culture medium supplemented with 10% DMSO.

**Pause Point:** The frozen vials can be kept in liquid nitrogen tanks long-term while genotyping the individual clones for correct reporter insertion (see below).

55. During either of the cell split points above (24- to 12-well or 12- to 6-well) or when freezing cells for cryopreservation, set aside some cells to isolate genomic DNA using the DNeasy Blood & Tissue Kit following manufacturer’s instructions.

***Note:*** If many clones are to be screened, it is desirable to extract genomic DNA as early as possible (split from 24- to 12-well) to process for genotyping in order to exclude unwanted clones from further culturing and expansion. When more cells are used for genomic DNA extraction, it will take a longer time for cells to grow back in the 12-wells or you might need to split cells into a smaller well format. It is possible to use alternative genomic DNA extraction kits such as the Epicentre QuickExtract DNA Extraction Solution which requires lower cell input. Although suitable for quick initial genotyping with short and easy-to-amplify PCR products, the quality and yield of DNA obtained from this method are consistently low in our hands and it is difficult to use the resulting products for more demanding genotyping involving challenging PCR conditions (see below).

**Figure 5:**
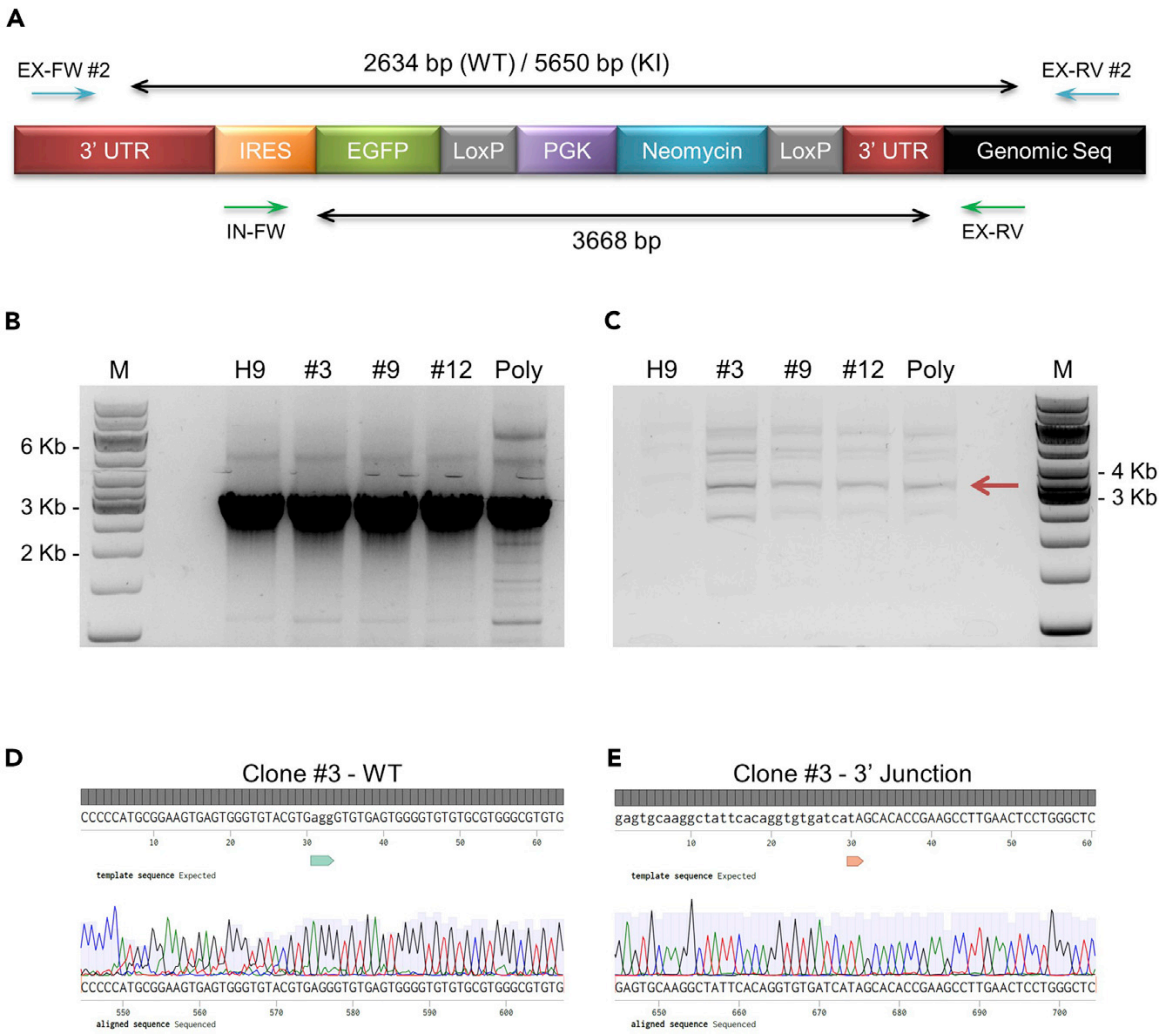
Genotyping of Clones before Antibiotic Cassette Removal (A) Schematic showing the *PAX7* allele after reporter knockin while retaining the antibiotic resistance cassette. Positions of genotyping primers and expected PCR product sizes are also shown. WT, wild type; KI, knockin; EX, external; IN, internal; FW, forward; RV, reverse. (B) Genotyping PCR products of the WT allele (EX-FW #2 and EX-RV #2). Note that the whole length of inserted reporter cassette was not successfully amplified in this case. M, molecular marker; Poly, the leftover “polyclonal” cells after single clone picking. (C) Similar to (B) with 3′ junctional genotyping PCR (IN-FW and EX-RV). (D) Sanger sequencing results showing the intact WT allele sequences surrounding PAM of the targeting gRNA (lower case bases denoted by the green arrow). (E) Sanger sequencing of the 3′ junctional region demonstrating proper reporter insertion at the intended locus. The right HA on the HDR vector is denoted by lower case bases while the genomic sequences outside the HA by upper case bases. The red arrow indicates the 3′ junction. In (D) and (E), chromatograms are shown with light gray peaks representing base call qualities. Dark gray bars on top indicate matches between expected and obtained DNA sequences.

56. Perform genotyping to assess if clones have the correct genomic insertion of the reporter cassette. Design PCR primers outside the HAs to amplify the wildtype (WT) locus to determine whether heterozygous integration takes place. Design primers for junctional PCR to amplify sequences encompassing part of the internal portion of the reporter cassette as well as a genomic region outside one of the HAs (Figure 5A and Table 6). Troubleshooting 2

**Table 6:** Genotyping PCR Primers

Primer Name	Primer Sequence
*Geno primer external forward #2*	TGGTCCCGATGGTGAAATGG
*Geno primer external reverse #2*	GTTTTAGGGCCAGGGGAACA
*Geno primer internal forward*	AATAAGGCCGGTGTGCGTTT
*Geno primer external reverse*	CAGGAGTTCAAGGCTTCGGT
*Geno primer internal reverse #2*	AACAGACCTTGCATTCCTTT
*Geno primer AmpR forward #2*	ATGTGGCGCGGTATTATCCC
*Geno primer AmpR reverse #2*	TTGTTGCCGGGAAGCTAGAG

**Table:** 

Purpose of Primer Pair	Genotyping Primer
Heterozygous *vs.* homozygous integration	Geno primer external forward #2
	Geno primer external reverse #2
Insertion at intended genomic location (3′ junction)	Geno primer internal forward
	Geno primer external reverse

Components	Amount
Template genomic DNA	25–50 ng
Forward primer	200 nM
Reverse primer	200 nM
10× AccuPrime PCR Buffer II	2.5 μL
AccuPrime High Fidelity *Taq* DNA Polymerase	0.2 μL
H_2_O	Supplement to 25 μL
Total	25 μL

PCR Cycling Conditions			
Steps	Temperature	Time	Cycles
Initial denaturation	94°C	30 s	1
Denaturation	94°C	20 s	30
Annealing	57°C	20 s	
Extension	68°C	5 min	
Final extension	68°C	5 min	1
Hold	4°C	Forever

57. Run the entire PCR products on a 0.8% TAE agarose gel, excise the correct-sized bands, and extract DNA using the MinElute Gel Extraction Kit according to manufacturer’s instructions (Figures 5B and 5C).
58. Submit the purified PCR products for Sanger sequencing to confirm the integration of the reporter cassette at the intended genomic location and the intactness of the unintegrated WT allele (Figures 5D and 5E). Refer to the following table for sequencing primers for each PCR amplicons (see Table 3 for primer sequences). Troubleshooting 3

**Table:** 

PCR Amplicon	Sequencing Primer
WT *PAX7* allele	Seq primer *PAX7* L-HA
	Seq primer *PAX7* R-HA-R
Integration junction region 3′	Seq primer internal forward
	Seq primer EGFP-C
	Seq primer *PAX7* R-HA

59. We chose one of the heterozygous clones with the correct genotyping (clone #3) to continue with the rest of the protocol.

### Removal of Antibiotic Resistance Cassette from Reporter Cells

**Timing: 4–6 weeks**

Since the strong constitutive PGK promoter driving neomycin resistance might have unpredictable effects on the expression of nearby endogenous gene(s), it is ideal to remove the PGK-Neo portion of the integrated reporter cassette from the host genome. Since this region is flanked by two loxP sites, in this step we describe procedures to excise out this sequence by introducing the Tat-Cre recombinase into the cells followed by another round of single-cell cloning. We also illustrate genotyping of the resultant clones to confirm successful removal of the antibiotic resistance cassette.

***Alternatives:*** Cells can be transfected with plasmids or transduced with viruses expressing Cre to remove the antibiotic resistance cassette.

***Alternatives:*** Antibiotic cassette removal can be initiated immediately after cells recover from antibiotic selection without an initial round of single-cell cloning to save time on cell culture and genotyping. However, this might increase the number of false positive clones picked and the need to genotyping more clones. In addition, performing two rounds of single-cell cloning practically eliminate any possibilities of multi-clonality of the established reporter cell lines.

60. Thaw the chosen reporter clone (#3 in this case) and culture for 1–2 passages to let the cells recover. Make sure colonies show regular growth rate and morphology without spontaneous differentiation before proceeding to the next steps.
61. Once colonies reach ~70%–80% confluent, dissociate into single cells with TrypLE Express and seed them into Matrigel-coated tissue culture vessels with hPSC culture medium supplemented with 10 μM of Y-27632 at a few different densities: 6,250, 12,500, 25,000 and 50,000 per cm^2^.
62. The next day, change to hPSC culture medium supplemented with 200 μg/mL of recombinant Tat-Cre (a gift from Anjana Rao Lab). Monitor the cells every hour and replace with regular hPSC culture medium after 3 h of Tat-Cre exposure when ~50%–70% of cells are dead.

**CRITICAL:** From this point on, do not include G418 in the hPSC culture medium as cells will regain sensitivity to the antibiotic once the selection cassette is successfully removed.

63. During the first week or so after Tat-Cre exposure, the majority of cells will die while a few cells will survive and grow to small-sized individual colonies ready to be picked.

**CRITICAL:** It is important to use an optimal combination of cell seeding density and Tat-Cre dosage and exposure time to enable single colony picking later on. If the cell density is too low or Tat-Cre is overdosed (concentration too high or exposure time too long), no cells will survive. On the other hand, if cell density is too high or Tat-Cre is not adequately used, there will be too many surviving colonies which will be in contact with each other before becoming suitable in size for single colony picking. Moreover, it will cause a high proportion of picked clones to not have successful Cre mediated antibiotic cassette removal. In our hands, a seeding density of 25,000 cells/cm^2^ with 3 h of Tat-Cre exposure at 200 μg/mL works the best. We have also tried lower Tat-Cre concentrations at 40 or 80 μg/mL with for ~24 h exposure. While 80 μg/mL was too toxic and killed all cells across all seeding densities, 40 μg/mL with 50,000 cells/cm^2^ produced workable colonies for downstream usage.

***Alternatives:*** The Tat-Cre recombinant protein can also be obtained from commercial sources such as the TAT-CRE Recombinase from Millipore-Sigma. It should be used following manufacturer’s instructions and the principals described above.

**Figure 6:**
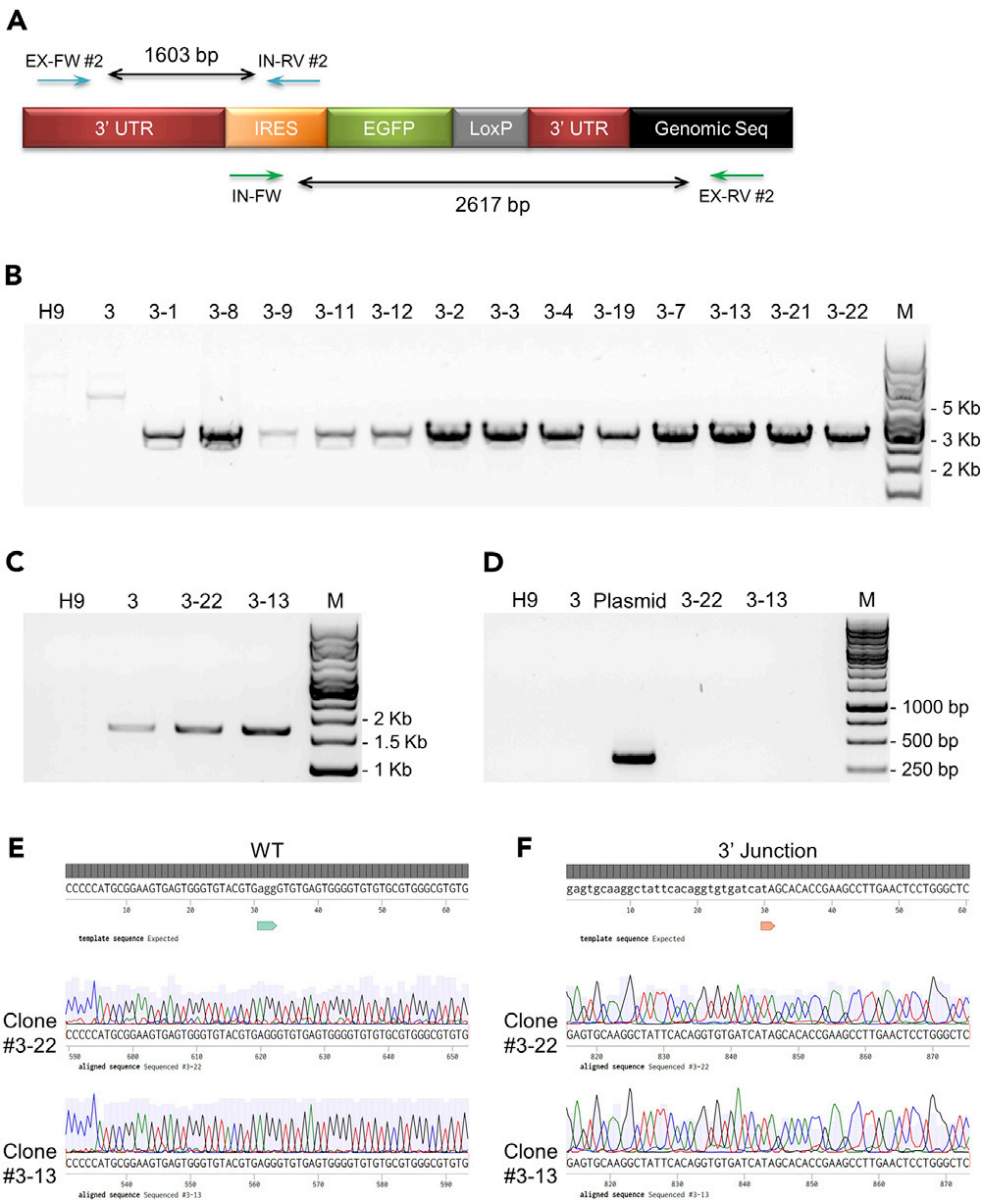
Genotyping of Clones after Antibiotic Cassette Removal (A) Schematic showing the *PAX7* knockin allele after antibiotic resistance cassette removal. Positions of genotyping primers and expected PCR product sizes are also listed except for those used to check donor vector backbone integration. EX: external; IN: internal; FW: forward; RV: reverse. (B) Genotyping PCR products of the 3′ junctional region (IN-FW and EX-RV #2) after successful antibiotic cassette removal. PCR products from the parental H9 cells as well as clone #3 containing the antibiotic cassette are included as controls. (C) Genotyping of 5′ junctional region (EX-FW #2 and IN-RV #2). (D) Genotyping to confirm no random vector backbone integration using AmpR forward #2 and reverse #2 genotyping primers. The HDR donor plasmid is included as a positive PCR control showing amplification of a region within the ampicillin resistant (AmpR) cassette in the vector backbone with an expected size of 345 bp. In (B)–(D), M indicates a molecular marker. (E) Sanger sequencing results showing the intact WT allele sequences surrounding PAM of the targeting gRNA (lower case bases denoted by the green arrow). (F) Sanger sequencing of the 3′ junctional region demonstrating proper reporter insertion at the intended locus. The right HA on the HDR vector is denoted by lower case bases while the genomic sequences outside the HA by upper case bases. The red arrow indicates the 3′ junction. In (E) and (F), chromatograms are shown with light gray peaks representing base call qualities. Dark gray bars on top indicate matches between expected and obtained DNA sequences.

64. Establish single clones, store cells and obtain genomic DNA for genotyping using similar procedures as described in steps 49–55 in section “Establishment of Reporter Cell Lines with Antibiotic Resistance.” During one of the passages of the clonal lines, set duplicate receiving wells and culture one well of cells with regular hPSC culture medium while the other with medium supplemented with 100 μg/mL of G418. Discard the clones which are still resistant to G418.
65. Perform genotyping to confirm clones with the antibiotic resistance cassette successfully removed. At this stage, we also performed genotyping PCRs to confirm the 5′ integration junction region and the absence of random plasmid backbone recombination (Figure 6 and Table 6).

**Table:** 

Purpose of Primer Pair	Genotyping Primer
Antibiotic cassette removal	Geno primer internal forward
	Geno primer external reverse #2
Insertion at intended genomic location (5′ junction)	Geno primer external forward #2
	Geno primer internal reverse #2
Random recombination of vector backbone	Geno primer AmpR forward #2
	Geno primer AmpR reverse #2

Components	Amount
Template genomic DNA or plasmid	25–50 ng (genomic DNA) or 5 ng (plasmid)
Forward primer	200 nM
Reverse primer	200 nM
10× AccuPrime PCR Buffer II (genomic DNA) Or 10× AccuPrime PCR Buffer I (plasmid)	2.5 μL
AccuPrime High Fidelity *Taq* DNA Polymerase	0.2 μL
H_2_O	Supplement to 25 μL
Total	25 μL

PCR Cycling Conditions			
Steps	Temperature	Time	Cycles
Initial denaturation	94°C	30 s	1
Denaturation	94°C	20 s	30
Annealing	58, 55 or 60°C^a^	20 s	
Extension	68°C	5, 2, or 0.5 min^a^	
Final extension	68°C	5 min	1
Hold	4°C	Forever	

a Annealing temperature of 58°C and extension time of 5 min for the primer pair to check antibiotic cassette removal. Annealing temperature of 55°C and extension time of 2 min for the primer pair to check insertion at intended genomic location (5′ junction region). Annealing temperature of 60°C and extension time of 0.5 min for the primer pair to check random recombination of vector backbone.

66. Run the entire PCR products on TAE agarose gels, excise the correct-sized bands and extract DNA (excluding those for vector backbone recombination examination) using the MinElute Gel Extraction Kit according to manufacturer’s instructions.
67. Submit the purified PCR products for Sanger sequencing to confirm the final integration of only the desired portion of the reporter cassette (Figures 6E and 6F). Refer to the following table for sequencing primers for each PCR amplicons (see Table 3 for primer sequences).

68. We chose clone #3-13 and #3-22 to continue with the rest of the protocol.

***Note:*** Pick the clones that show normal pluripotent colony morphology with comparable growth rate to the parental cells. Confirm pluripotency by checking marker expression such as OCT4, NANOG, and SOX2. Also confirm normal karyotypes of the selected clones before proceeding to the next steps.

66. Run the entire PCR products on TAE agarose gels, excise the correct-sized bands and extract DNA (excluding those for vector backbone recombination examination) using the MinElute Gel Extraction Kit according to manufacturer’s instructions.
67. Submit the purified PCR products for Sanger sequencing to confirm the final integration of only the desired portion of the reporter cassette (Figures 6E and 6F). Refer to the following table for sequencing primers for each PCR amplicons (see Table 3 for primer sequences).

**Table:** 

PCR Amplicon	Sequencing Primer
Reporter without the antibiotic cassette	Seq primer internal forward
	Seq primer external reverse #2
Integration junction region 5′	Seq primer external forward #2
	Seq primer *PAX7* L-HA
	Seq primer internal reverse #2

68. We chose clone #3-13 and #3-22 to continue with the rest of the protocol.

***Note:*** Pick the clones that show normal pluripotent colony morphology with comparable growth rate to the parental cells. Confirm pluripotency by checking marker expression such as OCT4, NANOG, and SOX2. Also confirm normal karyotypes of the selected clones before proceeding to the next steps.

### Functional Validation of Reporter Cells - Artificial *PAX7* Activation by CRISPR/dCas9-VPR

**Timing: ~2 weeks**

*PAX7* is expressed in skeletal muscle progenitor cells (SMPCs) and stem cells (satellite cells, SCs), as well as neural crest and a few other lineages (Jostes et al., 1990; Lepper and Fan, 2010). However, it is not expressed in undifferentiated hPSCs. Therefore, it is desired to be able to rapidly test the functionality of the GFP reporter to reflect endogenous *PAX7* expression. Here, we designed gRNAs targeting the *PAX7* promoter region coupled with the SP-dCas9-VPR system (Addgene, #63798) to artificially activate endogenous *PAX7* expression, followed by examination through fluorescent-activated cell sorting (FACS), immunofluorescent (IF) microscopy and quantitative real-time PCR (qPCR).

69. Construct the plasmids expressing gRNAs for *PAX7* activation/reporter validation according to steps 1–11 in section “Cloning of Candidate gRNA Expression Plasmids for Reporter Insertion.”
70. Screen for the most potent gRNA(s) to activate *PAX7* expression in the parental H9 hPSCs.
  a. Dissociate ~70%–80% confluent H9 colonies into single cells with TrypLE Express and seed them into Matrigel-coated tissue culture vessels with hPSC culture medium supplemented with 10 μM of Y-27632 at 25,000 cells per cm^2^.
  b. The next day, switch to presomitic mesoderm (PSM) promoting medium and culture the cells for 2 days with daily medium change.
  c. Two days later, dissociate into single cells with TrypLE Express and seed the cells into Matrigel-coated tissue culture vessels at 75,000 cells per cm^2^ with SC medium.
  d. One day after seeding, transfect the cells using ViaFect following manufacturer’s instructions. In each well on a 24-well plate, use 0.25 μg each of the dCas9-VPR and gRNA plasmid with 1.5 μL of ViaFect reagent (reagent-to-plasmid ratio of 3:1 (v/w)). Include a condition with dCas9-VPR plus empty gRNA cloning vector as a negative control. In addition to transfection of individual gRNA plasmids, set up a separate condition with all four gRNA plasmids mixed at an equal amount (0.0625 μg each).
  e. Culture the cells with daily SC medium change. Two days post transfection harvest the cells and extract RNA using the RNeasy Plus Micro Kit following manufacturer’s instructions.
  f. Generate cDNA using the iScript Reverse Transcription Supermix and perform qPCR to check *PAX7* expression using the SsoAdvanced Universal SYBR Green Supermix.

**Figure 7:**
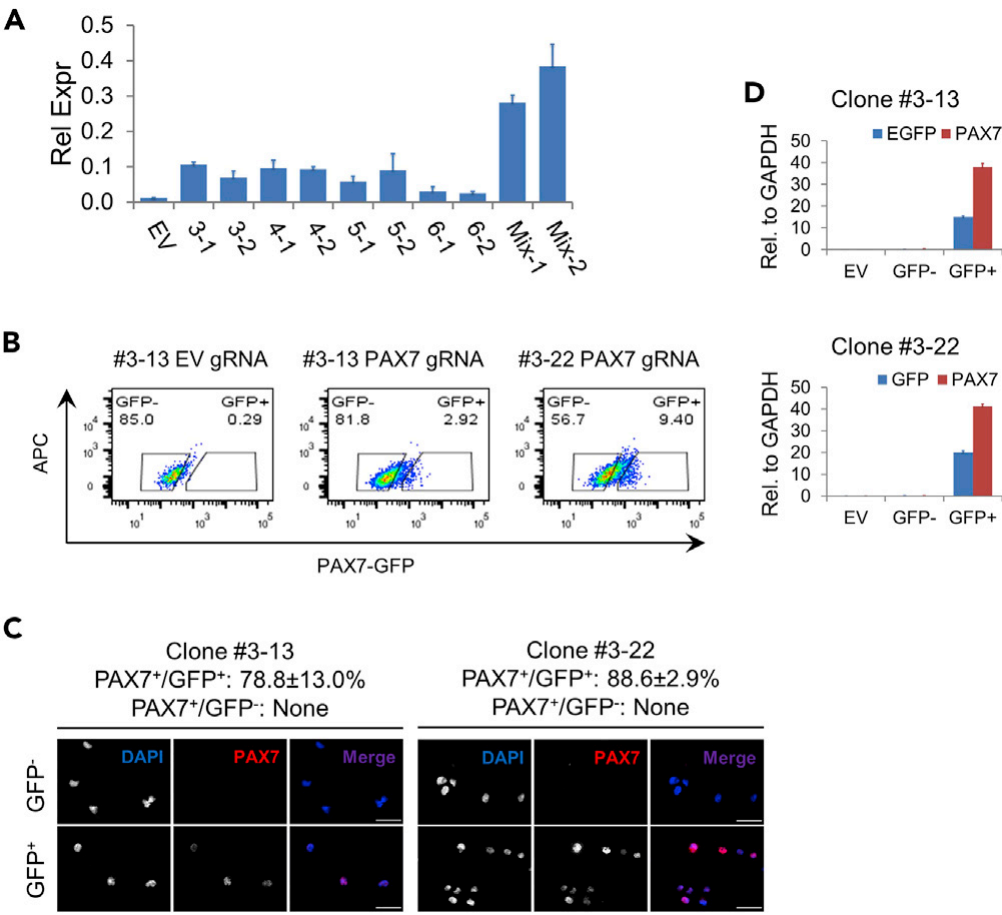
Artificial Reporter Validation through *PAX7* Promoter Activation Using the dCas9-VPR System (A) Pre-differentiated H9 cells were co-transfected with dCas9-VPR plus individual, mixed or empty (EV) gRNA plasmids. All transfections were performed in duplicates followed by RNA extraction. *PAX7* expression was examined by qPCR and normalized to *GAPDH*. (B) Reporter cells from clone #3-13 and #3-22 were transfected by dCas9-VPR along with the mixed *PAX7* promotor targeting or EV gRNA plasmids and sorted into GFP^+^ and GFP^−^ fractions. (C) Representative images of sorted cells subjected to cytospin followed by IF microscopy of PAX7. Numbers represent average percentage of sorted cells expressing PAX7 with SEM, from 2 independent experiments. Nuclei were counterstained by DAPI. Scale bars, 50 μm. (D) Sorted cells were examined by qPCR for *GFP* and *PAX7* expression with normalization to *GAPDH*. (B–D) Adapted from Supplemental Figure S4 in Xi et al. (2020).

**CRITICAL:** It is important to select gRNA(s) that can activate endogenous *PAX7* expression strong enough to enable the isolation of reporter (GFP) positive cells for downstream analysis. Consistent with reports from literature (Didovyk et al., 2016), we have found that the mixture of all four gRNAs resulted in a much higher *PAX7* expression level than any of the individual ones alone even when the total gRNA plasmids were kept at the same (Figure 7A). Accordingly, we transfected all four gRNAs in our further experiments for *PAX7* activation.

***Note:*** In our hands, the efficiency of simultaneous nucleofection of mid/large-sized plasmids into undifferentiated hPSCs is relatively low (although for small plasmids such as pmaxGFP, the efficiency can exceed 40%). This poses a challenge for the sensitivity of detection of *PAX7* activation when the plasmids do not carry features that can be used to enrich the transfected populations such as in our case. We have found in house that hPSCs differentiated to an early mesoderm stage are more easily transfected than the undifferentiated state (data not shown). Therefore, we employed this approach to enable comparison of *PAX7* activation by different gRNAs directly in unenriched populations.

***Alternatives:*** As long as sufficient transfection efficiency is achievable, undifferentiated hPSCs or hPSCs under a different state can be used to screen for *PAX7* activation gRNAs. Easy-to-transfect cells such as 293FT can also be used for this purpose.

71. Transfect the H9 reporter clones #3-13 and #3-22 with a mixture of plasmids encoding all four gRNAs along with dCas9-VPR following the same procedures as the above step 70. Use bigger culture vessels such as 6-well plates (with a total of 2.5 μg plasmids per well) for transfection to obtain enough cells for downstream processing.
72. On day 3 post transfection, dissociate into single cells with TrypLE Express and FACS sort each transfected clone into GFP^+^ and GFP^−^ fractions. Also obtain cells transfected with dCas9-VPR and the empty gRNA cloning vector as no *PAX7* activation controls (Figure 7B).
73. Load 10,000–20,000 cells in 100–200 μL volume into each channel of the Shandon Double Cytofunnel and attach the cells onto the Superfrost Plus Microscope Slides using a Cytospin 4 Cytocentrifuge at 1,000 rpm for 5 min. Fix the attached cells with 4% paraformaldehyde and proceed to stain for PAX7 protein following regular IF microscopy procedures. Image and quantify PAX7^+^ cells in GFP^+^ and GFP^−^ fractions (Figure 7C).
74. Extract RNA from the remaining cells not used for Cytospin and proceed with cDNA generation and qPCR to examine *PAX7* expression in sorted populations (Figure 7D).

### Functional Validation of Reporter Cells - Physiological *PAX7* Activation by Directed Skeletal Myogenic Differentiation

**Timing: ~5 weeks**

**Figure 8:**
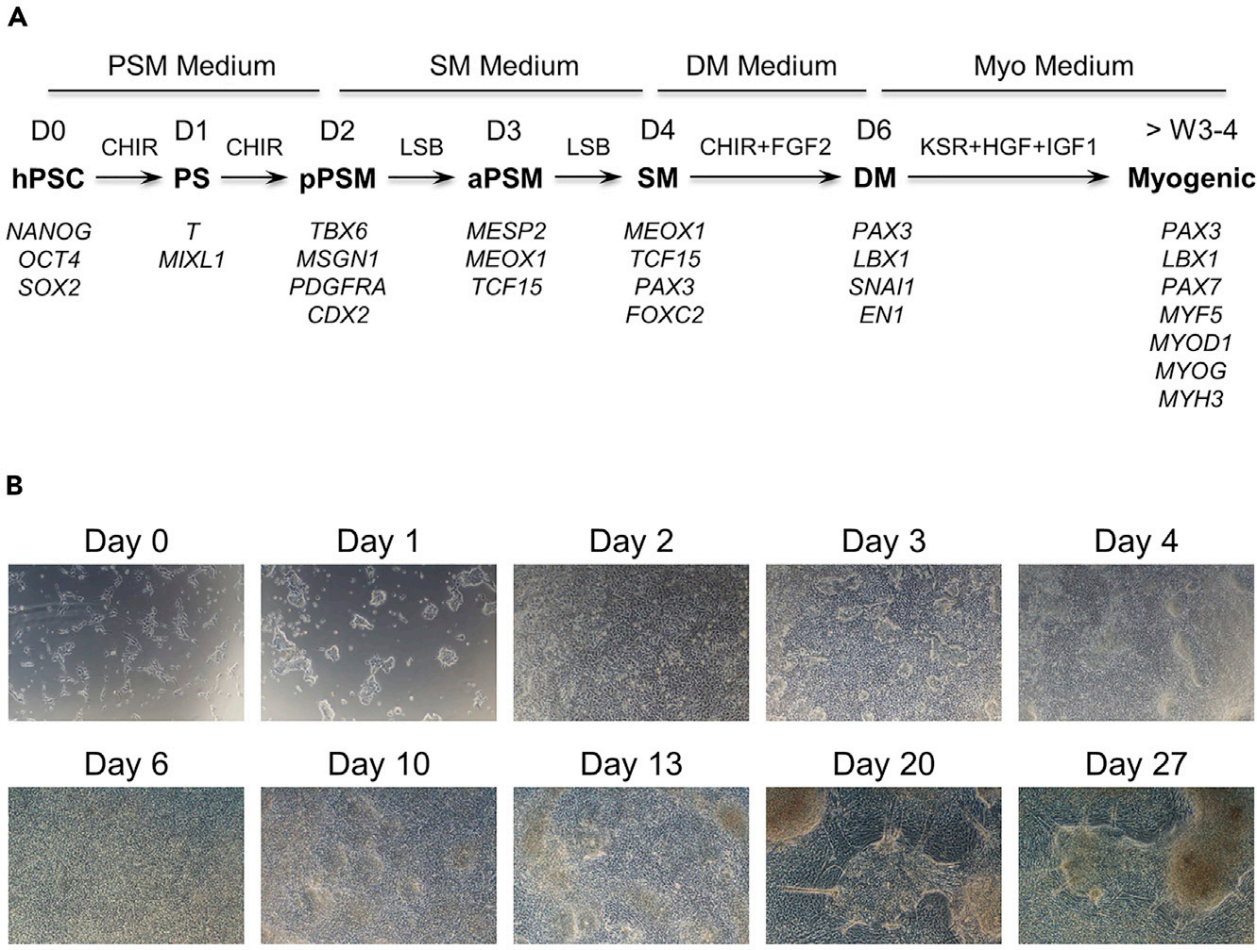
Overview of the Directed Myogenic Differentiation Protocol (A) Schematic of the protocol. PS, primitive streak; pPSM, posterior presomitic mesoderm; aPSM, anterior presomitic mesoderm; SM, somite; DM, dermomyotome; CHIR, CHIR99021; L, LDN-193189; SB, SB431542. (B) Morphology of differentiating cultures at key time points.

To examine the functionality of the *PAX7-GFP* reporter in a physiological context, we differentiated the reporter cells toward the skeletal muscle lineage as *PAX7* is highly expressed when cells are specified to a SMPC fate. We illustrate the procedures here using a chemically defined myogenic differentiation protocol we have previously established (Figure 8) (Xi et al., 2017). Other published protocols can also be used to achieve the same goal (Chal et al., 2015; Chal and Pourquie, 2017; Hicks et al., 2018; Magli and Perlingeiro, 2017; Shelton et al., 2014).

75. On day −1, dissociate ~70%–80% confluent reporter cell colonies into single cells with TrypLE Express and seed them onto Matrigel-coated tissue culture vessels with hPSC culture medium supplemented with 10 μM of Y-27632 at 12,500–25,000 cells per cm^2^.

***Note:*** Also set up a small-scale differentiation of the parental H9 cells to be used as gating controls for GFP FACS sorting at the end of the experiment.

**Figure 9:**
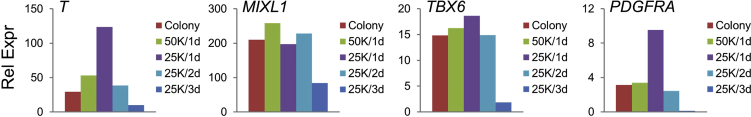
Influence of Cell Density on hPSC Early Stage Mesoderm Fate Specification H9 cells were seeded as single cells at 25,000 or 50,000 cells per cm^2^ and allowed to grow for 1–3 days before starting differentiation. Alternatively, cells were seeded as colonies and allowed to grow for 3 days to reach small-to-medium size before differentiation was initiated. Primitive streak (PS) markers *T* and *MIXL1* and posterior presomitic mesoderm (pPSM) markers *TBX6* and *PDGFRA* were examined by qPCR after 1 or 2 days, respectively. Expression was normalized to *GAPDH*.

**CRITICAL:** It is important to optimize the initial cell seeding densities for optimal differentiation outcomes. Too high of a seeding density will cause inadequate fate specification, whereas too low of a seeding density will result in excessive cytotoxicity during the initial differentiation stages. In addition, we have found that cells in a loosely aggregated state (1 day after single-cell seeding) are better specified compared to those in more compact colonies (multiple days after single-cell seeding or seeded as small colonies) (Figure 9). In our hands, a 12,500–25,000 cells per cm^2^ seeding density works well for H9 and derived reporter cells as well as some in-house generated iPSC lines (Young et al., 2016). However, seeding conditions might need to be further optimized for user-specific hPSC lines.

76. The next day (day 0), wash cells with PBS once and start differentiation by adding PSM promoting medium.
77. On day 1, cells are specified to a primitive streak (PS) fate. Refresh cells with PSM promoting medium.
78. On day 2, cells are specified to a posterior PSM (pPSM) fate. Wash cells with PBS once and add somite (SM) promoting medium.
79. On day 3, cells are specified to an anterior PSM (aPSM) fate. Refresh cells with SM promoting medium.
80. On day 4, cells are specified to a SM fate. Wash cells with PBS once and add dermomyotome (DM) promoting medium.

***Note:*** Cell fate change at these early stages are fairly rapid and cells can drift toward unwanted fates if they are not exposed to medium promoting the next desired state in time. Add SM promoting medium no more than 48 h after differentiation is initiated, and switch to DM promoting medium no more than 48 h after SM promoting medium is applied.

81. On day 5, refresh cells with DM promoting medium.
82. On day 6, cells are specified to a DM fate. Wash cells with PBS once and add myogenesis (Myo) promoting medium.
83. On day 8 and onwards, refresh cells with Myo promoting medium every other day until the end of differentiation. SMPCs emerge during week 3–4 of differentiation and the myogenic populations become robust after around week 4. Troubleshooting 4

***Note:*** It is normal to observe significant cell death during week 1–2 after switching to Myo promoting medium. Wash the wells 1–2 times with PBS to remove excessive dead cells and cellular debris at each medium change. The differentiating cells will recover after this period and become rapidly growing during the following week or so. If medium color is found to change to yellow too quickly before the next feeding schedule, refresh medium every day instead of every other day, or increase the volume of medium used (e.g., feed with 3 mL instead of regular 2 mL per 6-well). Cultures will become stable after week 3–4 of differentiation.

***Note:*** For differentiation longer than 5–6 weeks, it is not uncommon to see a sheet of differentiating cultures partially detached from the culture vessels. This sheet is mainly composed of dense extracellular matrix (ECM) produced during the differentiation process and the cells growing underneath it are still usable. Be careful when changing medium to avoid completely peeling the sheet off from the culture vessels.

**Figure 10:**
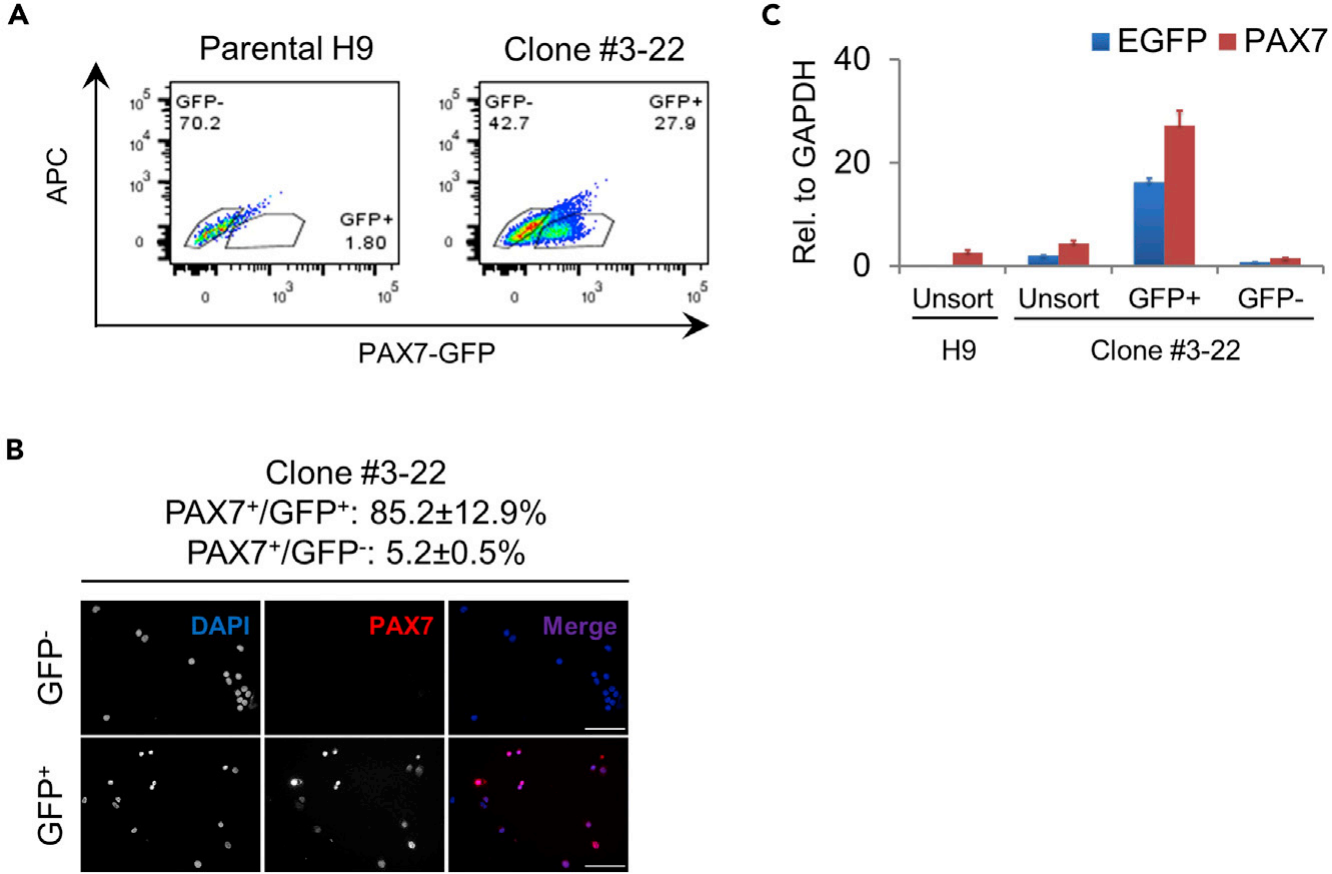
Physiological Reporter Validation through hPSC Directed Myogenic Differentiation (A) Representative FACS plots of clone #22 reporter cells under myogenic differentiated and sorted for GFP^+^ and GFP^−^ fractions. H9 parental cells from the same differentiation batch were used as GFP gating controls. (B) Representative images of sorted cells subjected to cytospin followed by IF microscopy of PAX7. Numbers represent average percentage of sorted cells expressing PAX7 with SEM, from 2 independent experiments. Nuclei were counterstained by DAPI. Scale bars, 100 μm. (C) Sorted clone #3-22 cells, as well as unsorted #3-22 or parental H9 cells, were examined by qPCR for *GFP* and *PAX7* expression with normalization to *GAPDH*. This figure is adapted from Supplemental Figure S4 in Xi et al. (2020).

84. After about 4 weeks, stop differentiation and harvest cells for functional reporter validation.
  a. Wash cells with PBS once and incubate at 37°C for ~5 min with 2 mg/mL of Collagenase IV in DMEM/F12 medium. After incubation, cultures will start to loosen up, but no cells should detach at this point.
  b. Remove the Collagenase IV solution using pipette tips (not vacuum) and wash once with PBS. Incubate at 37°C for 6–8 min with TrypLE Express. Tap the culture vessels occasionally to facilitate dissociation.
  c. Add FACS buffer at least 2 times the volume of TrypLE Express and triturate to fully dissociate the cultures.
    ***Note:*** The ECM present in the differentiation cultures tend to aggregate and trap cells during the dissociation process. Use pipette tips to “tear apart” the aggregated ECM before starting trituration. To prevent excessive clogging during pipetting, cut the end of tips or use wide-bore tips to triturate first followed by regular P1000 tips.
  d. Filter the cell solution through 100 μm cell strainers. Add additional volume of FACS buffer to wash the culture vessels and filter and combine with the cell solution.
  e. Filter the eluent again through 70 μm cell strainers, and centrifuge at 300 × *g* for 6 min.
  f. Resuspend the cell pellets with FACS buffer and count the cells. If necessary, adjust cell solution volume to achieve optimal cell concentrations for the specific cell sorters to be used.
    ***Optional:*** If desired, stain cells with antibodies of interest for FACS soring and/or flow cytometry analysis.
  g. Aliquot appropriate number of cells for sorting controls or samples into individual test tubes by filtering through 35 μm cell strainer snap caps.
  h. Add DAPI at a final concentration of 0.5–1 μg/mL to the sample and appropriate control tubes and mix well.
  i. FACS sort the reporter cells into GFP^+^ and GFP^−^ fractions.

85. Examine the sorted populations for PAX7 enrichment through Cytospin-IF microscopy and qPCR (Figure 10) as described in steps 73 and 74 in section “Functional Validation of Reporter Cells - Artificial PAX7 Activation by CRISPR/dCas9-VPR.”

## Expected Outcomes

After nucleofection of 800,000 H9 hPSCs and G418 selection in one well of a 6-well plate, we manually picked and transferred 19 individual colonies onto 24-wells. Some of the transferred colonies did not attach to and survive in the new wells, and some others showed aberrant growth kinetics or overt spontaneous differentiation. We discarded these clones and obtained 8 (~40%) remaining healthy clones. Five of these 8 clones (~60%) generated PCR genotyping products reflecting reporter cassette integration, while the remaining 3 are considered false positives from antibiotic selection. We performed more detailed genotyping on 3 of the 5 positive clones and found all of them were heterozygous for reporter insertion. We did not carry out further clone screening and genotyping as we prefer heterozygous reporter cells in our experiments to ensure one intact WT allele. However, homozygous knock-ins could be expected if more clones are screened.

We focused on one of the heterozygous clones (clone #3) and applied the Tat-Cre recombinase to remove the antibiotic resistance cassette. We manually picked and transferred 23 individual colonies onto 24-wells. We excluded 7 clones showing abnormal growth kinetics or excessive spontaneous differentiation, as well as 2 clones still resistant to G418. Of the remaining 14 clones (~60%), all of them generated genotyping PCR products representing successful antibiotic cassette removal from the inserted reporter. Based on the above rate of successful antibiotic cassette removal, picking a lower number of colonies (10 or less) could be sufficient to effectively obtain enough sub-clones for downstream processes.

Next, we selected two of the sub-clones (clone #3-13 and #3-22) to verify the proper reflection of *PAX7* expression by the inserted reporter (GFP). For rapid verification, we designed gRNAs targeting the *PAX7* promoter region and used the dCas9-VPR system to artificially activate endogenous *PAX7* expression. We found that co-transfection of all four gRNAs resulted in the most potent *PAX7* activation, and FACS sorted GFP^+^ cells were highly enriched for PAX7 transcript and protein.

In addition, we confirmed the function of the PAX7-GFP reporter using a physiological system where the reporter hPSCs were differentiated to generate PAX7-expressing SMPCs using our previously published protocol (Xi et al., 2017). This protocol generated ~20%–30% GFP^+^ cells, which were also enriched for PAX7.

## Limitations

We inserted IRES driven GFP at the 3′ end of the *PAX7* gene. This design allows *PAX7* and GFP to be expressed on the same transcript which enables regulation of GFP by the endogenous *PAX7* 3′ UTR. At the same time, the IRES element enables GFP and PAX7 to be translated as separate proteins, thus avoiding any potential functional alterations a fusion protein might possess. We also chose heterozygous knockin clones to ensure one intact WT allele in the reporter cells. Although the GFP^+^ cells derived from our reporter lines could be readily detected and isolated by FACS, the GFP signal is too weak to be easily discerned in dense differentiation cultures through the less sensitive fluorescent microscopy. This limits the use of our reporter cells from microscopy based PAX7 live imaging and high throughput screening. The weak GFP signal could be due to the longer *PAX7* transcript after insertion of IRES-GFP, which could potentially increase mRNA instability and decrease translational efficiency. Fusion of GFP to the C terminus of PAX7 while excluding its 3′ UTR will dramatically decrease the total transcript length (from ~6.2 to ~2.9 kb). In addition, the efficiency of IRES-mediated translation has been reported to be lower compared to the regular 5′ cap dependent mechanism and could be susceptible to preceding mRNA secondary structures (Hennecke et al., 2001). In this regard, insertion of a fluorescent reporter to the 5′ (Al Tanoury et al., 2020) or 3′ (Wu et al., 2016) of *PAX7* through the 2A self-cleaving peptides allows the endogenous translational machinery to drive more efficient reporter expression as standalone proteins. Moreover, using homozygous knockin cells could also increase GFP signals.

SMPCs express high levels of PAX7, but its expression rapidly decreases in committed myoblasts and disappears in terminally differentiated myocytes. Due to the high stability of GFP proteins (Eden et al., 2011; Li et al., 1998), cells that have previously expressed PAX7 could retain GFP while already having lost PAX7 proteins. Therefore, not all GFP^+^ sorted reporter cells are PAX7^+^. This is reflected by post-sort PAX7 Cytospin-IF microscopy and well-illustrated in singe cell RNA-seq studies from our and other groups (Al Tanoury et al., 2020; Xi et al., 2020). To enrich for 100% PAX7^+^ cells, variants of GFP or other fluorescent proteins with shorter half-lives (Andersen et al., 1998; Hackett et al., 2006; Li et al., 1998; Mateus and Avery, 2000) could be used to construct the reporter cells. Alternatively, novel cell surface markers might be identified to use in conjunction with the GFP reporter to maximally enrich for PAX7^+^ cells.

## Troubleshooting

### Problem 1

In “Secondary Screening of Candidate gRNAs in hPSCs”:

Cultures are over-confluent or too sparse after seeding 800,000 nucleofected hPSCs into one 24-well for 1 day.

### Potential Solution 1

In our experience, seeding nucleofected hPSCs at a high density greatly improves their viability. We have never experienced the issue of over-confluency when 800,000 cells were nucleofected with one or multiple CRISPR plasmids used in our studies (size range 4–11 kb). However, if your hPSC line is particularly tolerant to nucleofection-related stress or low amounts of small-sized plasmids are used, lower the number of cells to be nucleofected or decrease the seeding density post nucleofection to avoid over-confluency the next day.

On the other hand, if cells are very sparse 1-day post-nucleofection, optimize the nucleofection conditions to increase cell viability without sacrificing efficiency. Alternatively, skip passaging at day 1 and keep culturing the cells in the same 24-well (or split later on if necessary) until the end point of the experiment.

### Problem 2

In “Establishment of Reporter Cell Lines with Antibiotic Resistance”:

Designed genotyping primers do not amplify the whole inserted reporter cassette and/or genotyping PCR produces multiple unspecific bands.

### Potential Solution 2

PCR amplification of the long sequence encompassing the entire reporter cassette along with the two HAs is challenging. For genotyping of heterozygous clones, the primers will preferentially amplify the much shorter WT allele, which could lead to inadequate knockin allele amplification being detected. Try different primer pairs and PCR enzymes/conditions to improve amplification. If no satisfactory primers could be designed after extensive optimization, perform genotyping with 5′ and 3′ junctional PCR to cover the length of the entire insertion as well as confirm proper integration at the intended locus (as shown in our case).

There is an increased chance of non-specific amplification when genotyping PCR primers have to be designed in the extra-genic regions (as the case for our reverse external genotyping primers). Include proper controls such as parental cell line genomic DNA to distinguish specific *vs.* non-specific bands. Perform Sanger sequencing to confirm the correct PCR products.

### Problem 3

In “Establishment of Reporter Cell Lines with Antibiotic Resistance”:

All picked clones are false positives that do not show genotyping patterns of reporter integration.

### Potential Solution 3

It is not uncommon to have some false positive clones that survive the antibiotic selection while not having the reporter cassette inserted. However, if most or all of the picked clones are false positives, consider increasing the efficiency of CRISPR/Cas9 mediated HDR. This could be achieved by optimizing the nucleofection conditions for your particular hPSC lines or using a different transfection/electroporation system. You can also screen more gRNAs for those with higher DNA cutting efficiencies, which should mediate more HDR events in conjunction with Cas9.

### Problem 4

In “Functional Validation of Reporter Cells - Physiological *PAX7* Activation by Directed Skeletal Myogenic Differentiation”:

The directed differentiation protocol does not generate a significant number of myogenic cells.

### Potential Solution 4

Variations in differentiation efficiencies do exist among different research operators, experimental batches as well as hPSC lines, etc. In our experience, one of the most common reasons of failure is the inadequate specification of cells to a PSM and SM fate during the first 4 days of differentiation. In addition to the initial cell seeding density already discussed in the relevant section, the activation of WNT via CHIR99021 (CHIR) included in the PSM promoting medium for the first 2 days of differentiation is critical to proper cell fate specification. In our hands, CHIR concentrations ranging from 3 to 10 μM have been successfully used in our protocol for different hPSC lines. We highly recommend users to establish the optimal CHIR concentrations for their specific lines of interest. It is common to see obvious cell death after CHIR is applied but avoid using too high of a concentration that kills majority of the cells. As a general rule of thumb, ensure that at least 70%–80% of cells have adopted a PSM (TBX6^+^, CDX2^+^ or PDGFRα^+^KDR^−^) and SM (PAX3^+^, FOXC2^+^ or TCF15^+^) fate at day 2 and 4 of differentiation, respectively (Xi et al., 2017).

## Resource Availability

### Lead Contact

Further information and requests for resources and reagents should be directed to and will be fulfilled by the Lead Contact, April D. Pyle (apyle@mednet.ucla.edu).

### Materials Availability

Plasmids for reporter knockin and validation as well as H9 PAX7-GFP reporter cells generated in this study will be made available upon request.

### Data and Code Availability

This protocol does not generate or analyze any datasets or codes.
